# SPC-P1: a pathogenicity-associated prophage of *Salmonella paratyphi *C

**DOI:** 10.1186/1471-2164-11-729

**Published:** 2010-12-30

**Authors:** Qing-Hua Zou, Qing-Hai Li, Hong-Yun Zhu, Ye Feng, Yong-Guo Li, Randal N Johnston, Gui-Rong Liu, Shu-Lin Liu

**Affiliations:** 1 Department of Microbiology, Peking University Health Science Center, Beijing, PR China; 2 Genomics Research Center (one of The State-Province Key Laboratories of Biomedicine-Pharmaceutics of China), Harbin Medical University, Harbin, PR China; 3 Genetic Detection Center of First Hospital, Harbin Medical University, Harbin, PR China; 4 Department of Biochemistry and Molecular Biology, University of Calgary, Calgary, Canada; 5 Department of Microbiology and Infectious Diseases, University of Calgary, Calgary, Canada; 6 Systemomics Research Center, School of Pharmacy, Harbin Medical University, Harbin, PR China

## Abstract

**Background:**

*Salmonella paratyphi *C is one of the few human-adapted pathogens along with *S. typhi, S. paratyphi *A and *S. paratyphi *B that cause typhoid, but it is not clear whether these bacteria cause the disease by the same or different pathogenic mechanisms. Notably, these typhoid agents have distinct sets of large genomic insertions, which may encode different pathogenicity factors. Previously we identified a novel prophage, SPC-P1, in *S. paratyphi *C RKS4594 and wondered whether it might be involved in pathogenicity of the bacteria.

**Results:**

We analyzed the sequence of SPC-P1 and found that it is an inducible phage with an overall G+C content of 47.24%, similar to that of most *Salmonella *phages such as P22 and ST64T but significantly lower than the 52.16% average of the RKS4594 chromosome. Electron microscopy showed short-tailed phage particles very similar to the lambdoid phage CUS-3. To evaluate its roles in pathogenicity, we lysogenized *S. paratyphi *C strain CN13/87, which did not have this prophage, and infected mice with the lysogenized CN13/87. Compared to the phage-free wild type CN13/87, the lysogenized CN13/87 exhibited significantly increased virulence and caused multi-organ damages in mice at considerably lower infection doses.

**Conclusions:**

SPC-P1 contributes pathogenicity to *S. paratyphi *C in animal infection models, so it is possible that this prophage is involved in typhoid pathogenesis in humans. Genetic and functional analyses of SPC-P1 may facilitate the study of pathogenic evolution of the extant typhoid agents, providing particular help in elucidating the pathogenic determinants of the typhoid agents.

## Background

The bacterial genus *Salmonella *contains more than 2600 very closely related serovars, classified by the Kauffmann-White Scheme according to their differences in the somatic (O) and flagellar (H) antigens [1,2]. Although essentially all *Salmonella *bacteria are pathogens, they may have different host ranges or cause different diseases.

Over 1400 *Salmonella *serovars may infect humans, with most of them causing self-limiting gastroenteritis. On the other hand, a few *Salmonella *serovars, such as *Salmonella typhi, S. paratyphi *A, *S. paratyphi *B and *S. paratyphi *C, are adapted to humans and cause typhoid fever, a serious and potentially fatal systemic infection [3]. It is not clear whether these *Salmonella *typhoid agents use the same, similar or totally different pathogenic traits to infect the same host and cause the disease. Genomic comparisons between *S. typhi *and *S. paratyphi *A did not reveal a common genetic basis possibly responsible for human adaptation or typhoid pathogenesis [4,5]. Notably, various *Salmonella *pathogenicity islands (SPIs) or prophages have been identified in the *Salmonella *typhoid agents, e.g., SPI-7 in *S. typhi *[6,7] and *S. paratyphi *C [8], and SPA-1, SPA-2 and SPA-3 in *S. paratyphi *A [4], but their specific roles in typhoid pathogenesis have not been well established.

In a previous study, we located several insertions in the genome of *S. paratyphi *C strain RKS4594 by comparing it with other *Salmonella *genomes [9], including one, SPC-P1, which was a prophage present only in *S. paratyphi *C among all sequenced *Salmonella *strains [8]. In this study, we characterized this novel prophage, predicted its possible roles in the pathogenicity of *S. paratyphi *C, and evaluated its potential contributions to pathogenicity in animal experiments. We found that, although no previously known pathogenicity-associated genes were identified in the prophage, SPC-P1 did increase the pathogenicity of the bacteria.

## Results

### Genomic location and identification of prophage SPC-P1

We screened the complete nucleotide sequence of the *S. paratyphi *C RKS4594 genome (CP000857) by Phage_Finder http://phage-finder.sourceforge.net for possible prophage sequences and located five regions with typical prophage characteristics, with four of them having been reported in other *Salmonella *serovars and well studied previously, including Gifsy-1 and Gifsy-2 in *S. typhimurium *LT2 [10] and SPA-1 and Phage SPA-3-P2 in *S. paratyphi *A ATCC9150 [4]. These prophages have also been known to be present in several other *Salmonella *serovars, such as *S. choleraesuis *[11]. The remaining genomic region corresponds to the previously mapped 39 kb insertion between genes *purC *and *purF *[9] and has typical features of a prophage; here we designate this region SPC-P1. Sequence analysis showed that SPC-P1 lies between two adjacent genes, *pgtE *and *yfdC*, in *S. paratyphi *C RKS4594, whereas in fifteen other published *Salmonella *genomes (see their accession numbers below), we did not find DNA insertions in this region. The ends of SPC-P1 were set by two direct repeats of the sequence tggtgtcccctgcag, a typical feature for the ends of prophage DNA sequences. One of the repeat sequences begins at 109 bp upstream of SPC-P1 ORF1, and the other begins at 165 bp downstream of ORF53 and continues with an *arg *tRNA gene. The total length of SPC-P1 is 39,659 bp and the overall G+C content is 47.24%, which is similar to those of phage P22 (47.1%) [12,13] and ST64T (47.5%) [14] and is significantly lower than the 52.16% average of the *S. paratyphi *C RKS4594 chromosome.

### Layout and predicted products of SPC-P1 genes

Using Vector NTI 9.0 and GLIMMER3, we identified 53 ORFs in SPC-P1, designated consecutively from ORF1 through ORF53 (Additional file 1 Table S1), with the ORF encoding the terminase small unit as ORF1. As shown in Additional file 1 Table S1, the G+C contents of individual ORFs vary from 31.65% (ORF 16) to 53.89% (ORF 48), demonstrating an obvious mosaic structure.

Of the 53 ORFs, 47 had ATG and six (ORF13, ORF16, ORF21, ORF29, ORF31, ORF44) had GTG as the start codon. Functions of the SPC-P1genes were inferred based on similarities with characterized genes from other phages; some salient features of these protein-encoding genes are summarized in Additional file 1 Table S1.

**Figure 1:**
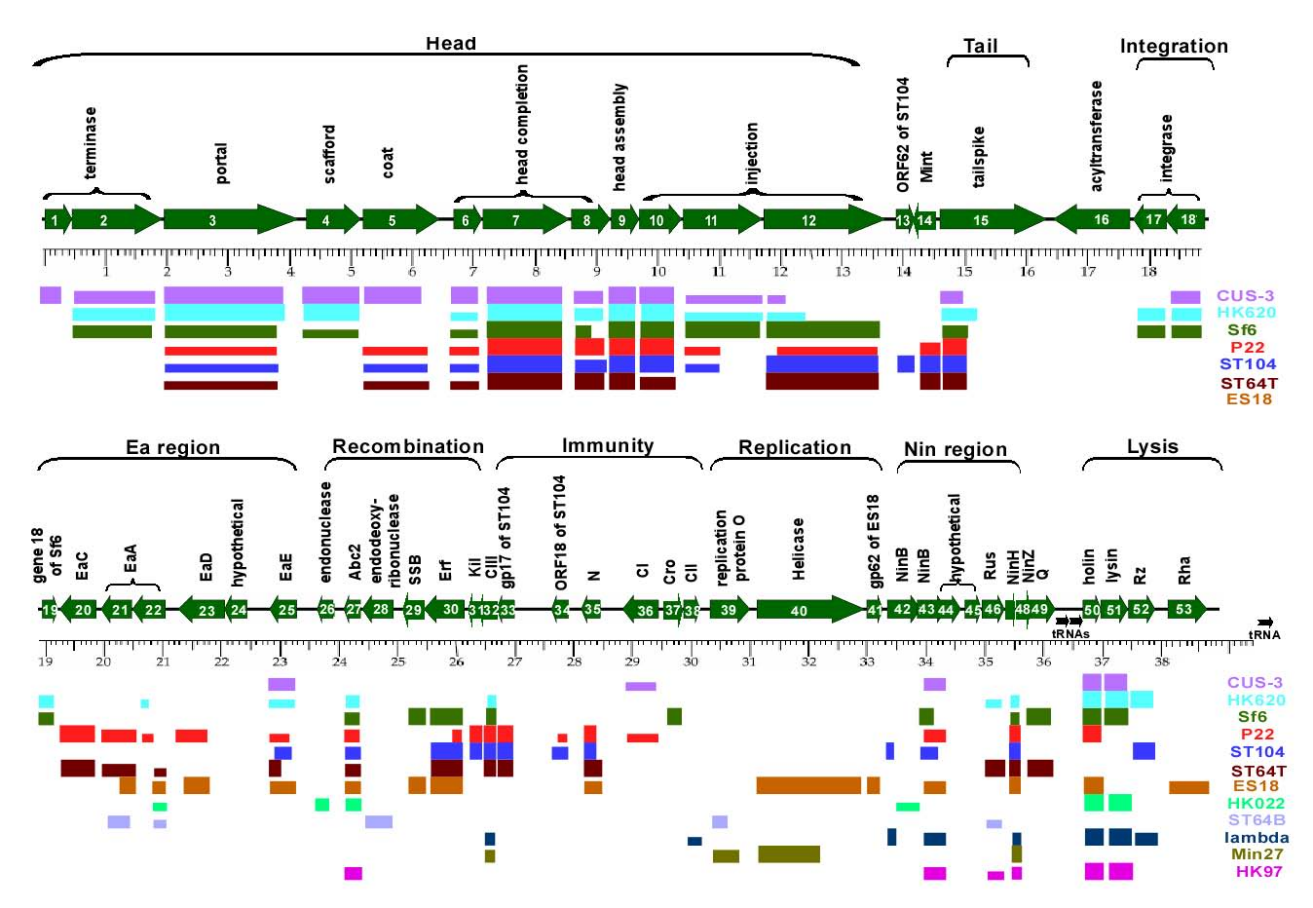
**Map of SPC-P1 (shown with a scale in kb)**. Green arrows above the scale represent predicted ORFs and their transcription directions. Selected gene names are shown within the arrows, and putative functions and names of homologous genes are given above. Below the scale, thinner colored boxes indicate the best matches with other phages. These matches vary from quite low to high identities; see Additional file 1 Table S1 for more details.

Three of the predicted SPC-P1 protein-encoding genes (ORF16, ORF24, and ORF45) have no phage-borne homologues in the current databases. However, it is worthwhile to note that the deduced product of ORF16 shows a low but significant level of similarity with the acyltransferase 3 of *Pseudomonas syringae py. Syringae *B728a (Additional file 1 Table S1). On the other hand, several bacteriophages such as *Shigella flexneri *bacteriophage Sf6 have O-antigen acetyltransferase gene in their genomes. As the protein products of such genes may alter the bacterial O-antigens [15], they likely would make important contributions to the bacterial virulence. The remaining 50 ORFs all have close homologues with previously characterized phages of enteric bacteria. The ORFs can be divided into nine main clusters according to the predicted functions, which are arranged in the following order: Head, Tail, Integration, Ea region, Recombination, Immunity, Replication, Nin region and Lysis (Figure 1). The gene cluster encoding the phage head covers a large region and consists of twelve ORFs. This region is closely related to the morphogenetic regions of previously characterized phages such as CUS-3, HK620, Sf6, P22, ST104 and ST64, however none of the latter six phages has the whole set of twelve ORFs seen in SPC-P1 (CUS-3, the most closely related phage, has the first eleven ORFs but only part of the twelfth ORF; Figure 1). The arrangement of the ORFs in this part of SPC-P1 is similar to that of CUS-3, Sf6 or HK620 in the gene order of 5'-small terminase-large terminase-portal-decoration-coat-3'. This suggests that ORF1-ORF12 define a type of head gene module, which is highly conservative in these phages (See more details in Additional file 1 Table S1). The gene cluster encoding phage tail is composed of only one ORF, ORF15, the deduced product of which shows certain similarities to those of some known phages, such as P22, ST104, ST64T, HK620, CUS-3 and Sf6. Additionally, SPC-P1 also contains gene clusters for phage integration (ORF17-18), recombination (ORF26-32), immunity (ORF33-38), replication (ORF39-41) and lysis (ORF50-53), suggesting that SPC-P1 encodes all functions required by and sufficient for an active phage.

### Phylogenetic analysis of SPC-P1

**Figure 2:**
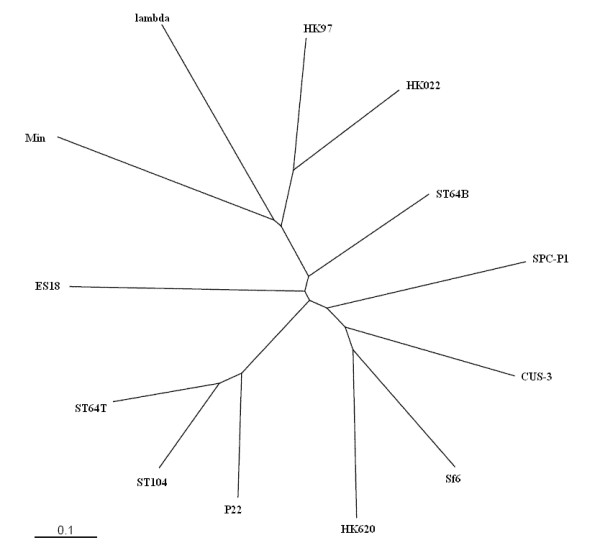
**Phylogenetic analysis of SPC-P1 with other phages by Mauve 2.3.1**.

Both nucleotide and deduced protein sequence homologies indicate that SPC-P1 is a member of the lambdoid phage group and the predicted functions of the genes are similar to those of lambdoid phages, especially to CUS-3, HK620, Sf6, ST64T, P22 and ST104. Each of these phages has about half of their deduced protein products showing high similarity with those of SPC-P1, providing evidence of mosaic architecture and extensive recombination events creating SPC-P1 during evolution. We chose twelve phages for comparisons with SPC-P1 using Mauve 2.3.1, which can produce a phylogenetic tree of mosaic phage and prophage genomes. The phylogenetic tree thus obtained revealed that SPC-P1 is closest to CUS-3 (Figure 2).

### Distribution of SPC-P1 in other *Salmonella *serovars and among *S. paratyphi *C strains

**Figure 3:**
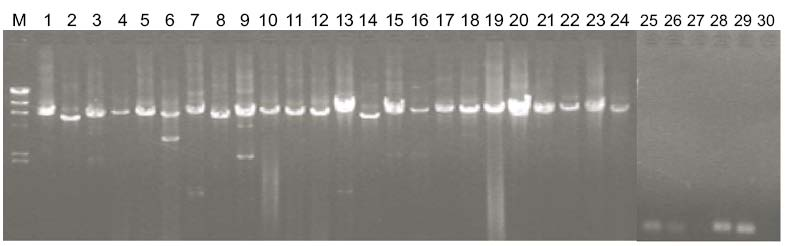
**Representative PCR results of SPC-P1 in *S. paratyphi *C strains**. Lanes: M, λ DNA/HindIII marker; 1-6, the six fragments of SPC-P1 in strain RKS4585; 7-12, the six fragments of SPC-P1 in strain RKS4586; 13-18, the six fragments of SPC-P1 in strain RKS4588; 19-24, the six fragments of SPC-P1 in strain RKS4594; 25-30, the results of CN13/87, which is negative for SPC-P1.

To determine whether SPC-P1 is present in other *Salmonella *serovars, we searched the GenBank Nucleotide collection (nr/nt) database and found that only some segments of SPC-P1 could be found in *S. choleraesuis *SC-B67 and *S. paratyphi *A ATCC9150. We then searched other wild type strains of *S. paratyphi *C for SPC-P1. Using primers amplifying six segments of SPC-P1 with overlapping regions (Table 1), we amplified regions of 407 to 7282 bp, 7217 to 13126 bp, 12813 to 19040 bp, 18726 to 25764 bp, 25409 to 32079 bp and 31704 to 38434 bp, respectively (Figure 3), and demonstrated that the whole sequence of SPC-P1 is also present in eight of the fourteen wild type strains tested (Table 2).

**Table 1:** Primers used for amplification of SPC-P1

primers	sequences	Site at SPC-P1	Product size (bp)
1 s	5'TTACTCCGCTGTTTCTTCTTCGTCTTCTTT 3'	407	6876
1 r	5'GGGATGGTGTGGGCTATGGTGCTTGCTTATGT 3'	7282	
2 s	5'ATCGTCTAGGCGTTTCATGATTGCTCTGTTCGTG 3'	7217	5910
2 r	5'GTTATGGTATTCAAACTCAATCCACTGTTGCTTA 3'	13126	
3 s	:5'GCGGCAAGGTGTGGAATGGAGAGCGAGAACTGGA 3'	12813	6228
3 r	5'ATTTTCCTTTGACGAGCACCCGACCACCATTATC 3'	19040	
4 s	5'GGAACGGTCAGAGAGATTGAGGTATGAGCAGAGT 3'	18726	7039
4 r	5'TTTTGGCATGATTCTGGCCTTCGATTCGATACGT 3'	25764	
5 s	5'AGCAACGCATGGCTAAGTGGGCAGAGGATAACGG 3'	25409	6671
5 r	5'TTAGCCGCATCAGAGCCAGGAACTGTCGGAAACG 3'	32079	
6 s	5'TCCGCAGCAATCAGGCAGGAGAAGCACTAACAGC 3'	31704	6731
6 r	5'ATGCAGCTGGCACACGTAAGCGGCGGGAC 3'	38434	

**Table 2:** PCR detection of SPC-P1 in S. paratyphi C strains

Strain No.	SGSC No.	PCR results
RKS4587	2289	-
RKS4620	2291	-
33K	2419	-
CN13/87	2712	-
RKS4585	2998	+
RKS4586	2999	+
RKS4588	3000	-
RKS4589	3001	+
RKS4590	3002	+
RKS4591	3003	+
RKS4592	3004	+
RKS4593	3005	-
RKS4595	3006	+
RKS4596	3007	-

"+"means the strain is positive for SPC-P1 and "-" means the strain is negative for SPC-P1

### Induction of SPC-P1 and morphological analysis

Since SPC-P1 seems to contain all necessary genes for a viable phage, we wondered whether it could be induced from the bacterial genome. Upon mitomycin C treatment, the culture of *S. paratyphi *C RKS4594 became clearer than the culture without mitomycin treatment, suggesting that phage were induced to lyse the cells.

**Figure 4:**
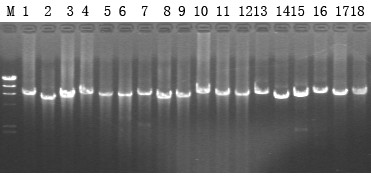
**PCR results of a plaque and two randomly picked CN13/87 colonies with SPC-P1 lysogenization**. Lanes: M, λ DNA/Hind III marker; 1-6, the six fragments of SPC-P1 in the genome of colony No.3; 7-12, the six fragments of SPC-P1 in the genome of colony No.4; 13-18, the six fragments of SPC-P1 in one plaque.

For propagation and characterization of the induced phage, we attempted finding a strain of *S. paratyphi *C that is sensitive to the phage. We used the lysate of RKS4594 after mitomycin C treatment to infect the seven *S. paratyphi *C strains, whose PCR results were negative for SPC-P1 (Table 2) and inspected plaque formation on them. We found plaques on strain CN13/87. To confirm that the plaques were formed by SPC-P1, we carried out PCR identification on the plaques; all six segments of SPC-P1 were amplified by PCR with the six pairs of primers (Figure 4 lanes 13-18).

**Figure 5:**
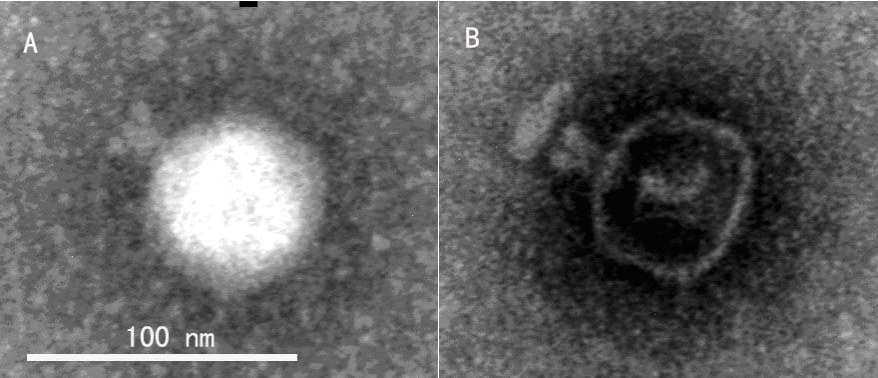
**SPC-P1 particle shown by transmission electron microscopy**. A, a phage particle with the head and short tail clearly seen; B, an empty phage head with a short tail connected to it.

Based on its genomic organization, we predicted that SPC-P1 phage would resemble CUS-3 with a characteristic short tail. To validate its morphology, we single-plaque isolated SPC-P1 and propagated the phage on CN13/87 for transmission electron microscopy. We saw typical short-tailed phage particles (Figure 5).

### Lysogenic conversion of CN13/87 by SPC-P1

**Figure 6:**
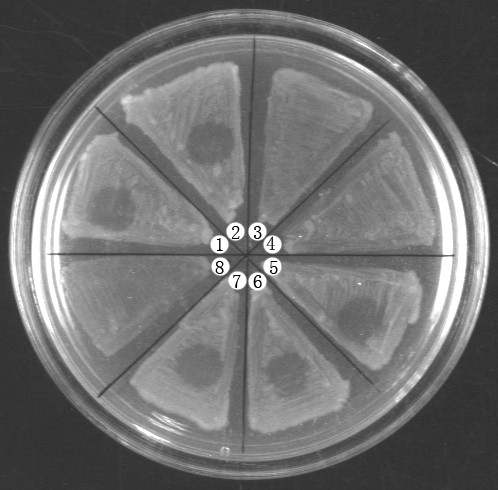
**Lysis resistance of lysogenized CN13/87 against SPC-P1**. Colonies: No. 1, *S. paratyphi *C CN13/87 wild type strain as the SPC-P1 negative control; No. 3, 4 and 8, lysogenized CN13/87 that are resistant to SPC-P1 lysis (SPC-P1 lysogenization confirmed by PCR); No. 2, 5-7, survivors of CN13/87 after SPC-P1 infection without lysogenization by the phage.

Since SPC-P1 could be induced from the genome of *S. paratyphi *C RKS4594 and strain CN13/87 is sensitive to SPC-P1, we wondered whether SPC-P1 could lysogenize CN13/87. SPC-P1 and CN13/87 were mixed at a multiplicity of infection of 1:100 and then co-cultured on LB plates. Single colonies were isolated and infected with SPC-P1 again. Three of the eight colonies, No. 3, 4 and 8 on the plate shown in Figure 6, were resistant to SPC-P1, suggesting lysogenization by, and immunity to, SPC-P1. We amplified all six segments of SPC-P1 from them, demonstrating lysogenization of the bacteria by SPC-P1; representative results are shown in Figure 4.

### Increased pathogenicity of lysogenized CN13/87 in mouse infection experiments

**Figure 7:**
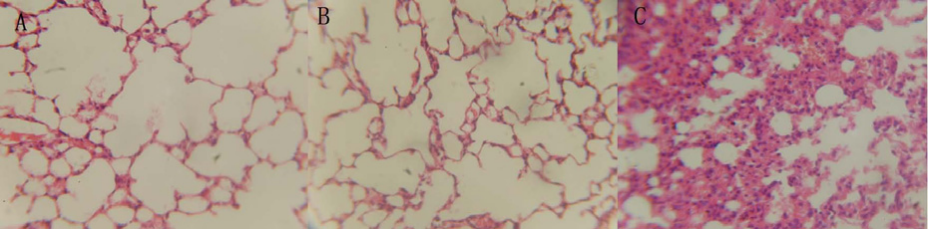
**Tissue damage of mice associated with SPC-P1-lysonenized CN13/87 infection**. Lung sections of mice orally inoculated with water (A), wild type CN13/87 at 10^9 ^cfu/0.5 ml (B) and SPC-P1-lysonenized CN13/87 at 10^9 ^cfu/0.5 ml (C). No significant differences were seen between (A) and (B), but inflammation cell infiltration and tissue damages are obvious in (C).

Since ORF16 shows certain sequence similarity with the acyltransferase 3 of *Pseudomonas syringae py. Syringae *B728a and the O-antigen genes play important roles in bacterial virulence, we wondered whether SPC-P1 might be involved in pathogenicity of the bacteria. We orally infected mice with 10^4^, 10^5^, 10^6^, 10^7^, 10^8 ^or 10^9 ^viable bacterial cells (colony forming units, cfu) of wild type or lysogenized CN13/87. We did not include the *S. paratyphi *C strain RKS4594, from which SPC-P1 was originally isolated, because we did not have a SPC-P1-free RKS4594 so comparison of virulence between SPC-P1-plus and SPC-P1-minus RKS4594 lines was not possible. On day 7 after infection, we sacrificed the mice and cultured homogenized liver, lung and spleen tissues to detect bacteria and determine the numbers of bacterial cells that will be required to establish infection in half of the inoculated mice (i.e., median infective dose, ID_50_). The wild type and lysogenized CN13/87 strains demonstrated greatly different pathogenicity on the animals, with ID_50 _values of 2.14 × 10^7 ^cfus and 6.76 × 10^4 ^cfus, respectively (Table 3). All recovered bacteria were confirmed to be identical to those in the inocula by serological and phage tests and pulsed field gel electrophoresis (data not shown). Pathological examinations showed multiple organ damages in mice infected with 10^9 ^cells of lysogenized CN13/87 (Figure 7).

**Table 3:** Infection^a^ of mice at different doses of bacteria

Group	Dose (cfu)	No. infected mice (%)	
		
		Lysogenized CN13/87	Wild type CN13/87
1	0	0	0

2	1 × 10^4^	1/6 (17%)	0

3	1 × 10^5^	4/6 (67%)	0

4	1 × 10^6^	5/6 (83%)	1/6 (17%)

5	1 × 10^7^	6/6 (100%)	2/6 (33%)

6	1 × 10^8^	6/6 (100%)	4/6 (67%)

7	1 × 10^9^	6/6 (100%)	6/6 (100%)

^a^ Infection means appearance of bacteria in any of the examined organ tissues of the animal.

## Discussion

Bacteria evolve by accumulating mutations and incorporating laterally transferred genes, among which phages are by far the most important driving force. For example, since the divergence from *E. coli *about 120-160 million years ago [16-18], *Salmonella *have developed into a great number of distinct lineages, with more than 2600 serovars currently recognized [2]. They all share a core genome, which is about 90% of the genes for *Salmonella *subgroup I serovars, with the remaining ca. 10% genes being specific to individual serovars [19]. Genomic analyses reveal that *Salmonella *harbor numerous temperate bacteriophages [13,20-23]. In fact, most of the non-core genome sequences are derived from phages, which play key roles in bacterial genome evolution and pathogenicity. In this study, we characterized a novel prophage, SPC-P1, in the genome of *S. paratyphi *C RKS4594 and demonstrated that this phage is present only in *S. paratyphi *C strains but not in any other *Salmonella *serovars tested. SPC-P1 exhibits typical characteristics of prophages, including a significantly lower overall G+ C content than that of the bacterial genome average, repeat sequences at the ends of its genome, and tRNA genes at the integration site. Sequence analysis showed that SPC-P1 has a substantial portion of its genome being highly related to previously characterized lambdoid phages and it has a complete set of genes to encode a viable phage. The mitomycin C induction test confirmed this.

Although prophages are widely found in bacterial genomes, most of them are defective, unable to produce viable phage particles. Sequence analysis indicated close relatedness of SPC-P1 to CUS-3 and electron microscopy also revealed morphological similarity between SPC-P1 and CUS-3. Like CUS-3, SPC-P1 also has a cosahedral head and a short tail. Since SPC-P1 could be induced from the bacterial genome and we had available the SPC-P1 sensitive strain CN13/87, we had the opportunity to propagate this phage for further studies. SPC-P1 could not only lyse CN13/87 but also lysogenize it, which allows us to study the possible roles of SPC-P1 in bacterial pathogenicity.

*S. paratyphi *C is one of the few *Salmonella *serovars that cause typhoid fever in humans, along with *S. typhi, S. paratyphi *A and *S. paratyphi *B, but it is not fully clear whether different *Salmonella *typhoid agents cause the disease by similar or distinct mechanisms. Prophages can contribute important biological properties to their bacterial hosts and analysis of the prophages may shed light on the evolution of their hosts. Considering that the human-adapted typhoid agents may have evolved by convergent processes [8], we speculate that *S. paratyphi *C may cause the disease by different mechanisms than those used by other *Salmonella *typhoid agents. As SPC-P1 is found only in *S. paratyphi *C, it may be involved in the pathogenesis of typhoid caused by *S. paratyphi *C. Taking the advantage that *S. paratyphi *C, unlike other human-adapted *Salmonella *typhoid agents, can infect hosts other than humans if large inocula are used [24,25], we compared pathogenicity of *S. paratyphi *C between SPC-P1-free and SPC-P1-lysogenized isogenic strains. We found that SPC-P1 significantly increased the pathogenicity of *S. paratyphi *C and caused multiple organ damages in the animals (see Table 3 and Figure 7), but the molecular basis is yet to be understood.

## Conclusions

SPC-P1 contributes pathogenicity to *S. paratyphi *C in animal infection models, so it is possible that this prophage is involved in typhoid pathogenesis in humans. Genetic and functional analyses of SPC-P1 may facilitate the study of evolution of the different typhoid agents, providing particular help in elucidating the pathogenic determinants of the typhoid agents.

## Methods

### Bacterial strains and growth conditions

Bacterial strains used in this study are listed in Table 2 and detailed information on them can be obtained from the *Salmonella *Genetic Stock Center http://www.ucalgary.ca/~kesander. Bacteria were grown overnight at 37°C with shaking in LB broth.

### Genome Sequencing

The genome sequence of RKS4594 was obtained from several pUC18 genomic shotgun libraries using dye terminator chemistry on Megabace1000 and ABI3730 automated sequencers as described previously [8].

### ORF prediction and homology search

Open reading frames (ORF) of SPC-P1 were predicted by Vector NTI and Glimmer 3. Products of ORFs were deduced based on homologies to known proteins by the BLASTP server of NCBI. Similarity of nucleotide sequence between SPC-P1 and the other completely sequenced *Salmonella *prophages was evaluated by the BLASTN server of NCBI.

### G+C content analysis and tRNA prediction

The G+C content of each predicted ORF was analyzed using the DNAstar software. tRNA was predicted using tRNAscan-SE software http://lowelab.ucsc.edu/tRNAscan-SE/.

### Distribution of SPC-P1 in other *S. paratyphi *C strains

Genomic DNA of *S. paratyphi *C strains was isolated by the Genomic DNA Extraction Kit (TIANZE, China). LA taq polymerases were purchased from Takara. PCR reactions were performed as follows: 94°C, 1 min; 98°C, 20 sec; 68°C, 10 min, 30 cycles; 72°C, 10 min.

#### Induction of SPC-P1

*S. paratyphi *C RKS4594 was grown in LB medium at 37°C for 4 h, followed by addition of four times volume of fresh LB. At this point, mitomycin C was added to a final concentration of 0.5 μ/ml. The cultures were shaken, 120r/min, at 37°C for 14 h. Chloroform was added to a final concentration of 1% to the culture, followed by vortex of the culture for 1 min. Cell debris was removed by centrifugation at 12000 rpm for 10 min. The supernatant containing SPC-P1 was preserved at 4°C until use.

### Plaque formation test

*S. paratyphi *C strain CN13/87 did not harbor SPC-P1 and so was used as a recipient in the test. Ten-fold serial dilutions of the bacterial lysate were made. Then 10 μl of a dilution and 100 μl of CN13/87 fresh culture (4 h) were mixed and incubated at 37°C for 20 min before addition of 3 ml 0.7% LB agar cooled to about 45°C. After mixing quickly, the 0.7% LB agar containing the lysate and bacteria was spread to a 1.5% LB agar plate, which then was cultured overnight. For identification, phage in individual plaques were picked up and propagated on CN13/87 before DNA was extracted for further PCR analysis and for transmission electron microscopy.

### Lysogenization of strain CN13/87

CN13/87 was cultured at 37°C in 2 ml LB for 4 h. Then 10 μl SPC-P1 (10^8 ^pfu/ml) was added and the culture was continued at 37°C overnight. Serial 10-fold dilutions of the culture were spread onto LB agar plates and incubated overnight at 37°C. Single colonies were picked up and spread uniformly onto fresh LB plates, eight colonies per plate. A small drop of SPC-P1 (about 5 μl) was placed onto the bacterial patches. The plates were cultured overnight before inspection of SPC-P1 plaques on the bacteria.

### Animal infection experiments

Female BALB/c mice, 6-8 weeks old, were divided into seven groups, twelve mice per group with six inoculated with wild type CN13/87 and six with SPC-P1-lysogenized CN13/87 orally with 0.5 ml sterile water containing no bacteria (group 1), 10^4 ^cfu/0.5 ml (group 2), 10^5 ^cfu/0.5 ml (group 3), 10^6 ^cfu/0.5 ml (group 4), 10^7 ^cfu/0.5 ml (group 5), 10^8 ^cfu/0.5 ml (group 6) or 10^9 ^cfu/0.5 ml (group 7). When the mice were sacrificed as specified, liver, lung and spleen tissues were taken for bacterial detection and histological examinations. ID50 was determined as described [26].

## Accession numbers

Genbank: *S. typhimurium *LT2 [NC_003197]; *S. choleraesuis *[SC-B67 NC_006905]; *S. paratyphi *A ATCC9150 [NC_006511]; *S. typhi *CT18 [NC_003198]; *S. typhi *Ty2 [NC_004631]; *S. paratyphi *C RKS4594 [CP000857]; *S. schwarzengrund *CVM19633 [NC_011094]; *S. paratyphi *A AKU_12601 [NC_011147]; *S. newport *SL254 [NC_011080]; *S. heidelberg *SL476 [NC_011083]; *S. gallinarum *287/91 [NC_011274]; *S. enteritidis *P125109 [NC_011294]; *S. dublin *CT_02021853 [NC_011205]; *S. agona *SL483 [NC_011149]; *S. arizonae *62:z4,z23:-- [NC_010067]; Enterobacteria phage ES18 [NC_006949]; Enterobacteria phage ST64T [NC_004348]; Enterobacteria phage ST104 [AB102868]; Enterobacteria phage CUS-3 [CP000711]; Enterobacteria phage HK620 [NC_002730]; Enterobacteria phage Sf6 [NC_005344]; Enterobacteria phage HK022[NC_002166]; Enterobacteria phage HK97 [NC_002167]; Enterobacteria phage lambda [NC_001416]; Bacteriophage P22[AF217253]; *Salmonella typhimurium phage *ST64B [AY055382]; Enterobacteria phage Min27 [NC_010237].

## Authors' contributions

QHZ initiated the project and carried out the bioinformatic analysis; QHL carried out animal experiments and determined ID_50 _of the bacteria on mice; QHZ and HYZ did phage induction, morphologic examination, lysogenization and PFGE experiments; YF was involved in bioinformatic analysis; YGL contributed reagents; RNJ, GRL and SLL coordinated the work; QHZ and SLL produced the manuscript. All authors read and approved the final manuscript.

## Supplementary Material

Additional file 1 **The ORFs in SPC-P1 DNA whose putative products exhibit significant homology to extant protein sequences**. This file includes the position of each ORFs in the chromosome of RKS4594, the start and stop codon, the size, %G+C, homology proteins and the % Identity range, E-value of each ORFs. Click here for file
