# Supplementary material for: SPC-P1: a pathogenicity-associated prophage of Salmonella paratyphi C
Source: BMC Genomics. 2010 Dec 30;11:729. doi: 10.1186/1471-2164-11-729 (PMC3022927; doi:10.1186/1471-2164-11-729)
Supplement: Additional file 1 — The ORFs in SPC-P1 DNA whose putative products exhibit significant homology to extant protein sequences. This file includes the position of each ORFs in the chromosome of RKS4594, the start and stop codon, the size, %G+C, homology proteins and the % Identity range, E-value of each ORFs. [file 1471-2164-11-729-S1.DOC]

**Table S1**. The ORFs in SPC-P1 DNA whose putative products exhibit significant homology to extant protein sequences

| ORF | Nucleotides | Condon | Size  (aa) | Strand | %G+C | E-value | %Identity/  range(aa) | Exhibits homology to (accession no. and/or reference) |
| --- | --- | --- | --- | --- | --- | --- | --- | --- |
|  | Start - end | start-stop |  |  |  |  |  |  |
| 1 | 1355472 1355915 | ATG TGA | 147 | + | 46.40 | 6e-64 | 90/131 | gb|ABQ88402.1|phage terminase small subunit [Enterobacteria phage CUS-3] |
| 2 | 1355912 1357387 | ATG TGA | 492 | + | 47.70 | 1e-179 | 65/488 | [gb|ABQ88401.1|](http://www.ncbi.nlm.nih.gov/entrez/query.fcgi?cmd=Retrieve&db=Protein&list_uids=148566126&dopt=GenPept&RID=HF7P61DS011&log$=protalign&blast_rank=1) phage terminase large subunit [Enterobacteria phage CUS-3] |
|  |  |  |  |  |  | 9e-170 | 63/488 | [ref\|NP_112076.1\|](http://www.ncbi.nlm.nih.gov/entrez/query.fcgi?cmd=Retrieve&db=Protein&list_uids=13559866&dopt=GenPept&RID=HF7P61DS011&log$=protalign&blast_rank=4) terminase large subunit [Enterobacteria phage HK620] |
|  |  |  |  |  |  | 8e-169 | 63/475 | [ref\|NP_958178.1\|](http://www.ncbi.nlm.nih.gov/entrez/query.fcgi?cmd=Retrieve&db=Protein&list_uids=41057280&dopt=GenPept&RID=HF7P61DS011&log$=protalign&blast_rank=5) putative large terminase subunit [Enterobacteria phage Sf6] |
| 3 | 1357392 1359587 | ATG TAA | 732 | + | 51.05 | 0.0 | 99/732 | [ref|YP_063721.1|](http://www.ncbi.nlm.nih.gov/entrez/query.fcgi?cmd=Retrieve&db=Protein&list_uids=51236733&dopt=GenPept&RID=SVXFYAJV015&log$=protalign&blast_rank=1) [ref|ZP_02682924.1|](http://www.ncbi.nlm.nih.gov/entrez/query.fcgi?cmd=Retrieve&db=Protein&list_uids=168260951&dopt=GenPept&RID=GZ1X00XJ016&log$=protalign&blast_rank=1) hypothetical protein SeH_A0877 [Salmonella enterica subsp. enterica serovar Hadar str. RI_05P066]  [GENE ID: 3991900 UTI89_C2653](http://www.ncbi.nlm.nih.gov/sites/entrez?db=gene&cmd=search&term=3991900&RID=GZ1X00XJ016&log$=geneexplicitprot&blast_rank=2) | bacteriophage HK620 gene 1-like |
|  |  |  |  |  |  | 0.0 | 97/726 | [gb\|ABQ88400.1\|](http://www.ncbi.nlm.nih.gov/entrez/query.fcgi?cmd=Retrieve&db=Protein&list_uids=148566125&dopt=GenPept&RID=GZ1X00XJ016&log$=protalign&blast_rank=2) phage portal protein [Enterobacteria phage CUS-3] |
|  |  |  |  |  |  | 0.0 | 55/709 | [gb\|ABA29409.1\|](http://www.ncbi.nlm.nih.gov/entrez/query.fcgi?cmd=Retrieve&db=Protein&list_uids=75906064&dopt=GenPept&RID=GZ1X00XJ016&log$=protalign&blast_rank=5) portal protein gp1 [Acyrthosiphon pisum bacteriophage APSE-2] |
|  |  |  |  |  |  | 0.0 | 55/679 | [ref\|NP_958179.1\|](http://www.ncbi.nlm.nih.gov/entrez/query.fcgi?cmd=Retrieve&db=Protein&list_uids=41057281&dopt=GenPept&RID=GZ1X00XJ016&log$=protalign&blast_rank=10) gene 3 protein [Enterobacteria phage Sf6] |
|  |  |  |  |  |  | 1e-103 | 34/727 | [GENE ID: 2944241 1](http://www.ncbi.nlm.nih.gov/sites/entrez?db=gene&cmd=search&term=2944241&RID=GZ1X00XJ016&log$=geneexplicitprot&blast_rank=12) \| portal protein [Enterobacteria phage P22] |
|  |  |  |  |  |  | 2e-103 | 34/728 | [ref\|YP_006406.1\|](http://www.ncbi.nlm.nih.gov/entrez/query.fcgi?cmd=Retrieve&db=Protein&list_uids=46358698&dopt=GenPept&RID=GZ1X00XJ016&log$=protalign&blast_rank=13) Gp1 [Enterobacteria phage ST104] |
|  |  |  |  |  |  | 3e-103 | 33/726 | [GENE ID: 955785 ST64Tp53](http://www.ncbi.nlm.nih.gov/sites/entrez?db=gene&cmd=search&term=955785&RID=GZ1X00XJ016&log$=geneexplicitprot&blast_rank=15) \| portal protein [Enterobacteria phage ST64T] |
| 4 | 1359681 1360571 | ATG TAA | 297 | + | 50.51 | 8e-170 | 100/297 | [GENE ID: 5590967 EcHS_A0311](http://www.ncbi.nlm.nih.gov/sites/entrez?db=gene&cmd=search&term=5590967&RID=GZ2322VG01R&log$=geneexplicitprot&blast_rank=1) | hypothetical protein [Escherichia coli HS] |
|  |  |  |  |  |  | 3e-169 | 99/297 | [gb\|ABQ88399.1\|](http://www.ncbi.nlm.nih.gov/entrez/query.fcgi?cmd=Retrieve&db=Protein&list_uids=148566124&dopt=GenPept&RID=GZ2322VG01R&log$=protalign&blast_rank=2) phage scaffold protein [Enterobacteria phage CUS-3]  [GENE ID: 3991899 UTI89_C2652](http://www.ncbi.nlm.nih.gov/sites/entrez?db=gene&cmd=search&term=3991899&RID=GZ2322VG01R&log$=geneexplicitprot&blast_rank=2) \| bacteriophage HK620 gene 8-like |
|  |  |  |  |  |  | 3e-14 | 32/220 | [ref\|NP_958180.1\|](http://www.ncbi.nlm.nih.gov/entrez/query.fcgi?cmd=Retrieve&db=Protein&list_uids=41057282&dopt=GenPept&RID=GZ2322VG01R&log$=protalign&blast_rank=6) gene 4 protein [Enterobacteria phage Sf6] |
|  |  |  |  |  |  | 3e-14 | 32/220 | [ref\|NP_112078.1\|](http://www.ncbi.nlm.nih.gov/entrez/query.fcgi?cmd=Retrieve&db=Protein&list_uids=13559868&dopt=GenPept&RID=GZ2322VG01R&log$=protalign&blast_rank=7) scaffold protein [Enterobacteria phage HK620] |
| 5 | 1360593 1361843 | ATG TAA | 417 | + | 50.92 | 0.0 | 99/417 | [gb|ABQ88398.1|](http://www.ncbi.nlm.nih.gov/entrez/query.fcgi?cmd=Retrieve&db=Protein&list_uids=148566123&dopt=GenPept&RID=GZ2F8ZSG011&log$=protalign&blast_rank=1) putative coat protein [Enterobacteria phage CUS-3] |
|  |  |  |  |  |  | 1e-31 | 29/423 | [ref\|NP_059630.1\|](http://www.ncbi.nlm.nih.gov/entrez/query.fcgi?cmd=Retrieve&db=Protein&list_uids=9635538&dopt=GenPept&RID=GZ2F8ZSG011&log$=protalign&blast_rank=7) coat protein [Enterobacteria phage P22] |
|  |  |  |  |  |  | 2e-31 | 29/423 | [ref\|NP_720329.1\|](http://www.ncbi.nlm.nih.gov/entrez/query.fcgi?cmd=Retrieve&db=Protein&list_uids=24371587&dopt=GenPept&RID=GZ2F8ZSG011&log$=protalign&blast_rank=8) 5 [Enterobacteria phage ST64T]  [ref\|YP_006408.1\|](http://www.ncbi.nlm.nih.gov/entrez/query.fcgi?cmd=Retrieve&db=Protein&list_uids=46358700&dopt=GenPept&RID=GZ2F8ZSG011&log$=protalign&blast_rank=8) Gp5 [Enterobacteria phage ST104] |
| 6 | 1362057 1362515 | ATG TGA | 153 | + | 48.58 | 3e-84 | 100/153 | [gb|ABQ88396.1|](http://www.ncbi.nlm.nih.gov/entrez/query.fcgi?cmd=Retrieve&db=Protein&list_uids=148566121&dopt=GenPept&RID=GZ4RK2ER01R&log$=protalign&blast_rank=1) putative head completion protein [Enterobacteria phage CUS-3] |
|  |  |  |  |  |  | 5e-26 | 41/143 | [ref\|NP_958183.1\|](http://www.ncbi.nlm.nih.gov/entrez/query.fcgi?cmd=Retrieve&db=Protein&list_uids=41057285&dopt=GenPept&RID=GZ4RK2ER01R&log$=protalign&blast_rank=5) gene 7 protein [Enterobacteria phage Sf6] |
|  |  |  |  |  |  | 5e-26 | 41/143 | [ref\|NP_112081.1\|](http://www.ncbi.nlm.nih.gov/entrez/query.fcgi?cmd=Retrieve&db=Protein&list_uids=13559871&dopt=GenPept&RID=GZ4RK2ER01R&log$=protalign&blast_rank=6) DNA stabilization protein [Enterobacteria phage HK620] |
|  |  |  |  |  |  | 7e-19 | 40/159 | [ref\|NP_059632.1\|](http://www.ncbi.nlm.nih.gov/entrez/query.fcgi?cmd=Retrieve&db=Protein&list_uids=9635540&dopt=GenPept&RID=GZ4RK2ER01R&log$=protalign&blast_rank=12) head completion protein [Enterobacteria phage P22] |
|  |  |  |  |  |  | 0.043 | 40/159 | [ref\|YP_006410.1\|](http://www.ncbi.nlm.nih.gov/entrez/query.fcgi?cmd=Retrieve&db=Protein&list_uids=46358702&dopt=GenPept&RID=GZ4RK2ER01R&log$=protalign&blast_rank=14) Gp4 [Enterobacteria phage ST104] |
|  |  |  |  |  |  | 8e-18 | 40/159 | [ref\|NP_720331.1\|](http://www.ncbi.nlm.nih.gov/entrez/query.fcgi?cmd=Retrieve&db=Protein&list_uids=24371589&dopt=GenPept&RID=GZ4RK2ER01R&log$=protalign&blast_rank=16) 4 [Enterobacteria phage ST64T] |
| 7 | 1362528 1363943 | ATG TAA | 472 | + | 50.92 | 0.0 | 99/472 | [ref|YP_215346.1|](http://www.ncbi.nlm.nih.gov/entrez/query.fcgi?cmd=Retrieve&db=Protein&list_uids=62178929&dopt=GenPept&RID=GZ4VEU9K01R&log$=protalign&blast_rank=1) DNA stabilization protein [Salmonella enterica subsp. enterica serovar Choleraesuis str. SC-B67] |
|  |  |  |  |  |  | 0.0 | 97/472 | [ref\|NP_112082.1\|](http://www.ncbi.nlm.nih.gov/entrez/query.fcgi?cmd=Retrieve&db=Protein&list_uids=13559872&dopt=GenPept&RID=GZ4VEU9K01R&log$=protalign&blast_rank=4) DNA stabilization protein [Enterobacteria phage HK620] |
|  |  |  |  |  |  | 0.0 | 97/472 | [gb\|ABQ88395.1\|](http://www.ncbi.nlm.nih.gov/entrez/query.fcgi?cmd=Retrieve&db=Protein&list_uids=148566120&dopt=GenPept&RID=GZ4VEU9K01R&log$=protalign&blast_rank=5) putative head completion protein [Enterobacteria phage CUS-3] |
|  |  |  |  |  |  | 0.0 | 97/472 | [ref\|NP_958184.1\|](http://www.ncbi.nlm.nih.gov/entrez/query.fcgi?cmd=Retrieve&db=Protein&list_uids=41057286&dopt=GenPept&RID=GZ4VEU9K01R&log$=protalign&blast_rank=6) gene 8 protein [Enterobacteria phage Sf6] |
|  |  |  |  |  |  | 0.0 | 94/472 | [ref\|NP_059633.1\|](http://www.ncbi.nlm.nih.gov/entrez/query.fcgi?cmd=Retrieve&db=Protein&list_uids=9635541&dopt=GenPept&RID=GZ4VEU9K01R&log$=protalign&blast_rank=9) head completion protein [Enterobacteria phage P22] |
|  |  |  |  |  |  | 0.0 | 93/472 | [ref\|NP_720332.1\|](http://www.ncbi.nlm.nih.gov/entrez/query.fcgi?cmd=Retrieve&db=Protein&list_uids=24371590&dopt=GenPept&RID=GZ4VEU9K01R&log$=protalign&blast_rank=10) 10 [Enterobacteria phage ST64T] |
|  |  |  |  |  |  | 0.0 | 92/472 | [ref\|YP_006412.1\|](http://www.ncbi.nlm.nih.gov/entrez/query.fcgi?cmd=Retrieve&db=Protein&list_uids=46358704&dopt=GenPept&RID=GZ4VEU9K01R&log$=protalign&blast_rank=15) gp10 [Enterobacteria phage ST104] |
| 8 | 1363950 1364585 | ATG TAA | 212 | + | 43.71 | 7e-117 | 99/212 | [ref|YP_215347.1|](http://www.ncbi.nlm.nih.gov/entrez/query.fcgi?cmd=Retrieve&db=Protein&list_uids=62178930&dopt=GenPept&RID=GZ4ZUBPX01R&log$=protalign&blast_rank=1) packaged DNA stabilization protein gp26 [Salmonella enterica subsp. enterica serovar Choleraesuis str. SC-B67] |
|  |  |  |  |  |  | 9e-111 | 93/212 | [ref\|NP_720333.1\|](http://www.ncbi.nlm.nih.gov/entrez/query.fcgi?cmd=Retrieve&db=Protein&list_uids=24371591&dopt=GenPept&RID=GZ4ZUBPX01R&log$=protalign&blast_rank=2) 26 [Enterobacteria phage ST64T]  [dbj\|BAF80724.1\|](http://www.ncbi.nlm.nih.gov/entrez/query.fcgi?cmd=Retrieve&db=Protein&list_uids=157734718&dopt=GenPept&RID=GZ4ZUBPX01R&log$=protalign&blast_rank=2) head completion protein [Enterobacteria phage P22] |
|  |  |  |  |  |  | 2e-61 | 57/233 | [ref\|YP_006413.1\|](http://www.ncbi.nlm.nih.gov/entrez/query.fcgi?cmd=Retrieve&db=Protein&list_uids=46358705&dopt=GenPept&RID=GZ4ZUBPX01R&log$=protalign&blast_rank=10) gp26 [Enterobacteria phage ST104] |
|  |  |  |  |  |  | 2e-59 | 55/233 | [gb\|ABQ88394.1\|](http://www.ncbi.nlm.nih.gov/entrez/query.fcgi?cmd=Retrieve&db=Protein&list_uids=148566119&dopt=GenPept&RID=GZ4ZUBPX01R&log$=protalign&blast_rank=11) putative head completion protein [Enterobacteria phage CUS-3] |
|  |  |  |  |  |  | 8e-58 | 54/233 | [ref\|NP_112083.1\|](http://www.ncbi.nlm.nih.gov/entrez/query.fcgi?cmd=Retrieve&db=Protein&list_uids=13559873&dopt=GenPept&RID=GZ4ZUBPX01R&log$=protalign&blast_rank=12) DNA stabilization protein [Enterobacteria phage HK620] |
|  |  |  |  |  |  | 3e-32 | 53/128 | [ref\|NP_958185.1\|](http://www.ncbi.nlm.nih.gov/entrez/query.fcgi?cmd=Retrieve&db=Protein&list_uids=41057287&dopt=GenPept&RID=GZ4ZUBPX01R&log$=protalign&blast_rank=15) gene 9 protein [Enterobacteria phage Sf6] |
| 9 | 1364588 1365040 | ATG TAG | 151 | + | 50.33 | 1e-85 | 100/151 | [ref|YP_215348.1|](http://www.ncbi.nlm.nih.gov/entrez/query.fcgi?cmd=Retrieve&db=Protein&list_uids=62178931&dopt=GenPept&RID=GZ56CP5201R&log$=protalign&blast_rank=1) hypothetical protein SC0361 [Salmonella enterica subsp. enterica serovar Choleraesuis str. SC-B67] |
|  |  |  |  |  |  | 2e-84 | 98/151 | [ref\|YP_006414.1\|](http://www.ncbi.nlm.nih.gov/entrez/query.fcgi?cmd=Retrieve&db=Protein&list_uids=46358706&dopt=GenPept&RID=GZ56CP5201R&log$=protalign&blast_rank=3) gp14 [Enterobacteria phage ST104] |
|  |  |  |  |  |  | 2e-83 | 97/151 | [ref\|NP_720334.1\|](http://www.ncbi.nlm.nih.gov/entrez/query.fcgi?cmd=Retrieve&db=Protein&list_uids=24371592&dopt=GenPept&RID=GZ56CP5201R&log$=protalign&blast_rank=5) 14 [Enterobacteria phage ST64T] |
|  |  |  |  |  |  | 3e-83 | 97/151 | [ref\|NP_112084.1\|](http://www.ncbi.nlm.nih.gov/entrez/query.fcgi?cmd=Retrieve&db=Protein&list_uids=13559874&dopt=GenPept&RID=GZ56CP5201R&log$=protalign&blast_rank=6) head assembly protein [Enterobacteria phage HK620] |
|  |  |  |  |  |  | 3e-83 | 97/151 | [gb\|ABQ88393.1\|](http://www.ncbi.nlm.nih.gov/entrez/query.fcgi?cmd=Retrieve&db=Protein&list_uids=148566118&dopt=GenPept&RID=GZ56CP5201R&log$=protalign&blast_rank=7) putative head assembly protein [Enterobacteria phage CUS-3] |
|  |  |  |  |  |  | 1e-82 | 95/151 | [ref\|NP_958186.1\|](http://www.ncbi.nlm.nih.gov/entrez/query.fcgi?cmd=Retrieve&db=Protein&list_uids=41057288&dopt=GenPept&RID=GZ56CP5201R&log$=protalign&blast_rank=10) gene 10 protein [Enterobacteria phage Sf6] |
|  |  |  |  |  |  | 2e-82 | 95/151 | [ref\|YP_063716.1\|](http://www.ncbi.nlm.nih.gov/entrez/query.fcgi?cmd=Retrieve&db=Protein&list_uids=51236729&dopt=GenPept&RID=GZ56CP5201R&log$=protalign&blast_rank=11) virion stability factor [Enterobacteria phage P22] |
| 10 | 1365046 1365738 | ATG TAA | 231 | + | 52.38 | 4e-100 | 95/209 | [ref|NP_958187.1|](http://www.ncbi.nlm.nih.gov/entrez/query.fcgi?cmd=Retrieve&db=Protein&list_uids=41057289&dopt=GenPept&RID=GZ58XUJ601R&log$=protalign&blast_rank=1) gene 11 protein [Enterobacteria phage Sf6] |
|  |  |  |  |  |  | 2e-86 | 82/211 | [gb\|ABQ88392.1\|](http://www.ncbi.nlm.nih.gov/entrez/query.fcgi?cmd=Retrieve&db=Protein&list_uids=148566117&dopt=GenPept&RID=GZ58XUJ601R&log$=protalign&blast_rank=7) putative phage injection protein [Enterobacteria phage CUS-3] |
|  |  |  |  |  |  | 6e-8 | 88/189 | [ref\|NP_112085.1\|](http://www.ncbi.nlm.nih.gov/entrez/query.fcgi?cmd=Retrieve&db=Protein&list_uids=13559875&dopt=GenPept&RID=GZ58XUJ601R&log$=protalign&blast_rank=8) DNA transfer protein [Enterobacteria phage HK620] |
|  |  |  |  |  |  | 2e-76 | 83/189 | [ref\|YP_063717.1\|](http://www.ncbi.nlm.nih.gov/entrez/query.fcgi?cmd=Retrieve&db=Protein&list_uids=51236730&dopt=GenPept&RID=GZ58XUJ601R&log$=protalign&blast_rank=9) injection protein [Enterobacteria phage P22] |
|  |  |  |  |  |  | 7e-76 | 82/189 | [ref\|YP_006415.1\|](http://www.ncbi.nlm.nih.gov/entrez/query.fcgi?cmd=Retrieve&db=Protein&list_uids=46358707&dopt=GenPept&RID=GZ58XUJ601R&log$=protalign&blast_rank=12) gp7 [Enterobacteria phage ST104] |
|  |  |  |  |  |  | 3e-50 | 66/197 | [ref\|NP_720335.1\|](http://www.ncbi.nlm.nih.gov/entrez/query.fcgi?cmd=Retrieve&db=Protein&list_uids=24371593&dopt=GenPept&RID=GZ58XUJ601R&log$=protalign&blast_rank=20) 7 [Enterobacteria phage ST64T] |
| 11 | 1365751 1367043 | ATG TAA | 431 | + | 49.03 | 0.0 | 97/431 | [ref|YP_215320.1|](http://www.ncbi.nlm.nih.gov/entrez/query.fcgi?cmd=Retrieve&db=Protein&list_uids=62178903&dopt=GenPept&RID=SVW7Z42M01R&log$=protalign&blast_rank=1) [ref|NP_958188.1|](http://www.ncbi.nlm.nih.gov/entrez/query.fcgi?cmd=Retrieve&db=Protein&list_uids=41057290&dopt=GenPept&RID=GZ5EW3FC01R&log$=protalign&blast_rank=1)gene 12 protein [Enterobacteria phage Sf6] |
|  |  |  |  |  |  | 2e-140 | 62/423 | [ref\|NP_706180.1\|](http://www.ncbi.nlm.nih.gov/entrez/query.fcgi?cmd=Retrieve&db=Protein&list_uids=24111670&dopt=GenPept&RID=GZ5EW3FC01R&log$=protalign&blast_rank=4) putative prophage DNA injection protein [Shigella flexneri 2a str. 301] |
|  |  |  |  |  |  | 1e-54 | 50/239 | [f\|NP_059637.1\|](http://www.ncbi.nlm.nih.gov/entrez/query.fcgi?cmd=Retrieve&db=Protein&list_uids=9635545&dopt=GenPept&RID=GZ5EW3FC01R&log$=protalign&blast_rank=12) injection protein [Enterobacteria phage P22] |
|  |  |  |  |  |  | 1e-51 | 34/462 | [ref\|NP_112086.1\|](http://www.ncbi.nlm.nih.gov/entrez/query.fcgi?cmd=Retrieve&db=Protein&list_uids=13559876&dopt=GenPept&RID=GZ5EW3FC01R&log$=protalign&blast_rank=13) DNA transfer protein [Enterobacteria phage HK620] |
|  |  |  |  |  |  | 5e-51 | 33/462 | [gb\|ABQ88391.1\|](http://www.ncbi.nlm.nih.gov/entrez/query.fcgi?cmd=Retrieve&db=Protein&list_uids=148566116&dopt=GenPept&RID=GZ5EW3FC01R&log$=protalign&blast_rank=14) putative phage injection protein [Enterobacteria phage CUS-3] |
|  |  |  |  |  |  | 3e-41 | 44/243 | [ref\|YP_006416.1\|](http://www.ncbi.nlm.nih.gov/entrez/query.fcgi?cmd=Retrieve&db=Protein&list_uids=46358708&dopt=GenPept&RID=GZ5EW3FC01R&log$=protalign&blast_rank=20) gp20 [Enterobacteria phage ST104] |
| 12 | 1367046 1369040 | ATG TAG | 665 | + | 50.53 | 0.0 | 93/665 | [ref|NP_958189.1|](http://www.ncbi.nlm.nih.gov/entrez/query.fcgi?cmd=Retrieve&db=Protein&list_uids=41057291&dopt=GenPept&RID=GZ5H7YBY01R&log$=protalign&blast_rank=1) gene 13 protein [Enterobacteria phage Sf6] |
|  |  |  |  |  |  | 0.0 | 80/666 | [ref\|NP_720337.1\|](http://www.ncbi.nlm.nih.gov/entrez/query.fcgi?cmd=Retrieve&db=Protein&list_uids=24371595&dopt=GenPept&RID=GZ5H7YBY01R&log$=protalign&blast_rank=5) 16 [Enterobacteria phage ST64T]  [ref\|YP_006417.1\|](http://www.ncbi.nlm.nih.gov/entrez/query.fcgi?cmd=Retrieve&db=Protein&list_uids=46358709&dopt=GenPept&RID=GZ5H7YBY01R&log$=protalign&blast_rank=5) gp16 [Enterobacteria phage ST104] |
|  |  |  |  |  |  | 5e-65 | 34/570 | [gb\|AAF75055.1\|](http://www.ncbi.nlm.nih.gov/entrez/query.fcgi?cmd=Retrieve&db=Protein&list_uids=8439633&dopt=GenPept&RID=GZ5H7YBY01R&log$=protalign&blast_rank=11) DNA transfer protein [Enterobacteria phage P22 |
|  |  |  |  |  |  | 2e-19 | 47/137 | [gb\|ABQ88390.1\|](http://www.ncbi.nlm.nih.gov/entrez/query.fcgi?cmd=Retrieve&db=Protein&list_uids=148566115&dopt=GenPept&RID=GZ5H7YBY01R&log$=protalign&blast_rank=20) putative DNA transfer protein [Enterobacteria phage CUS-3] |
|  |  |  |  |  |  | 6e-19 | 36/256 | [ref\|NP_112087.1\|](http://www.ncbi.nlm.nih.gov/entrez/query.fcgi?cmd=Retrieve&db=Protein&list_uids=13559877&dopt=GenPept&RID=GZ5H7YBY01R&log$=protalign&blast_rank=21) DNA transfer protein [Enterobacteria phage HK620] |
| 13 | 1369173 1369472 | GTG TGA | 100 | + | 43.67 | 3e-49 | 98/99 | [ref|YP_006418.1|](http://www.ncbi.nlm.nih.gov/entrez/query.fcgi?cmd=Retrieve&db=Protein&list_uids=46358710&dopt=GenPept&RID=GZ5NYUS101R&log$=protalign&blast_rank=1) ORF62 [Enterobacteria phage ST104] |
| 14 | 1369173 1369472 | ATG TAG | 82 | - | 42.57 | 1e-39 | 100/82 | [ref|NP_720338.1|](http://www.ncbi.nlm.nih.gov/entrez/query.fcgi?cmd=Retrieve&db=Protein&list_uids=24371596&dopt=GenPept&RID=T8DYA3WZ013&log$=protalign&blast_rank=1) Mnt [Enterobacteria phage ST64T]  [ref|YP_006419.1|](http://www.ncbi.nlm.nih.gov/entrez/query.fcgi?cmd=Retrieve&db=Protein&list_uids=46358711&dopt=GenPept&RID=T8DYA3WZ013&log$=protalign&blast_rank=1) Mint [Enterobacteria phage ST104]  [ref|YP_151582.1|](http://www.ncbi.nlm.nih.gov/entrez/query.fcgi?cmd=Retrieve&db=Protein&list_uids=56414507&dopt=GenPept&RID=T8DYA3WZ013&log$=protalign&blast_rank=1) regulatory [Salmonella enterica subsp. enterica serovar Paratyphi A str. ATCC 9150] |
|  |  |  |  |  |  | 8e-23 | 60/81 | [ref\|NP_059641.1\|](http://www.ncbi.nlm.nih.gov/entrez/query.fcgi?cmd=Retrieve&db=Protein&list_uids=9635548&dopt=GenPept&RID=T8DYA3WZ013&log$=protalign&blast_rank=3) Mnt [Enterobacteria phage P22] |
| 15 | 1369880 1371628 | ATG TGA | 583 | + | 41.51 | 0.0 | 74/587 | [ref|YP_215354.1|](http://www.ncbi.nlm.nih.gov/entrez/query.fcgi?cmd=Retrieve&db=Protein&list_uids=62178937&dopt=GenPept&RID=GZCW85EE016&log$=protalign&blast_rank=1) hypothetical protein SC0367 [Salmonella enterica subsp. enterica serovar Choleraesuis str. SC-B67] |
|  |  |  |  |  |  | 2e-58 | 94/117 | [ref\|ZP_02665057.1\|](http://www.ncbi.nlm.nih.gov/entrez/query.fcgi?cmd=Retrieve&db=Protein&list_uids=168240125&dopt=GenPept&RID=GZCW85EE016&log$=protalign&blast_rank=2) phage P22 tailspike protein |
|  |  |  |  |  |  | 4e-58 | 94/117 | [gb\|AAY43005.1\|](http://www.ncbi.nlm.nih.gov/entrez/query.fcgi?cmd=Retrieve&db=Protein&list_uids=66220002&dopt=GenPept&RID=GZCW85EE016&log$=protalign&blast_rank=3) tail protein [Salmonella enterica phage A18a] |
|  |  |  |  |  |  | 2e-57 | 95/117 | [ref\|YP_006420.1\|](http://www.ncbi.nlm.nih.gov/entrez/query.fcgi?cmd=Retrieve&db=Protein&list_uids=46358712&dopt=GenPept&RID=GZCW85EE016&log$=protalign&blast_rank=4) gp9 [Enterobacteria phage ST104] |
|  |  |  |  |  |  | 2e-57 | 94/117 | [gb\|ABN47328.1\|](http://www.ncbi.nlm.nih.gov/entrez/query.fcgi?cmd=Retrieve&db=Protein&list_uids=125631922&dopt=GenPept&RID=GZCW85EE016&log$=protalign&blast_rank=5) Gp9 [Salmonella phage SETP14] |
|  |  |  |  |  |  | 2e-57 | 94/117 | [gb\|AAY43004.1\|](http://www.ncbi.nlm.nih.gov/entrez/query.fcgi?cmd=Retrieve&db=Protein&list_uids=66219986&dopt=GenPept&RID=GZCW85EE016&log$=protalign&blast_rank=8) tail protein [Salmonella enterica phage A1] |
|  |  |  |  |  |  | 2e-57 | 94/117 | [ref\|NP_720339.1\|](http://www.ncbi.nlm.nih.gov/entrez/query.fcgi?cmd=Retrieve&db=Protein&list_uids=24371597&dopt=GenPept&RID=GZCW85EE016&log$=protalign&blast_rank=10) 9 [Enterobacteria phage ST64T] |
|  |  |  |  |  |  | 4e-47 | 51/205 | [ref\|NP_112090.1\|](http://www.ncbi.nlm.nih.gov/entrez/query.fcgi?cmd=Retrieve&db=Protein&list_uids=13559880&dopt=GenPept&RID=GZCW85EE016&log$=protalign&blast_rank=18) tail spike protein [Enterobacteria phage HK620] |
|  |  |  |  |  |  | 2e-46 | 72/126 | [gb\|ABQ88383.1\|](http://www.ncbi.nlm.nih.gov/entrez/query.fcgi?cmd=Retrieve&db=Protein&list_uids=148566108&dopt=GenPept&RID=GZCW85EE016&log$=protalign&blast_rank=19) endosialidase tailspike protein [Enterobacteria phage CUS-3] |
|  |  |  |  |  |  | 2e-45 | 71/121 | [gb\|ABA54611.1\|](http://www.ncbi.nlm.nih.gov/entrez/query.fcgi?cmd=Retrieve&db=Protein&list_uids=76781668&dopt=GenPept&RID=GZCW85EE016&log$=protalign&blast_rank=22) tailspike protein [Bacteriophage e34] |
|  |  |  |  |  |  | 1e-42 | 70/120 | [ref\|NP_958190.1\|](http://www.ncbi.nlm.nih.gov/entrez/query.fcgi?cmd=Retrieve&db=Protein&list_uids=41057292&dopt=GenPept&RID=GZCW85EE016&log$=protalign&blast_rank=25) gene 14 protein [Enterobacteria phage Sf6] |
| 16 | 1371698 1372948 | GTG TAA | 417 | - | 31.65 | 8e-34 | 29/384 | [ref|ZP_03283074.1|](http://www.ncbi.nlm.nih.gov/entrez/query.fcgi?cmd=Retrieve&db=Protein&list_uids=209908590&dopt=GenPept&RID=GZCYH4JX016&log$=protalign&blast_rank=1) hypothetical protein ENTCAN_02866 [Enterobacter cancerogenus ATCC 35316] |
|  |  |  |  |  |  | 2e-32 | 30/381 | [ref\|YP_233812.1\|](http://www.ncbi.nlm.nih.gov/entrez/query.fcgi?cmd=Retrieve&db=Protein&list_uids=66043971&dopt=GenPept&RID=GZCYH4JX016&log$=protalign&blast_rank=2) acyltransferase 3 [Pseudomonas syringae pv. syringae B728a] |
| 17 | 1372987 1373532 | ATG TAA | 182 | - | 48.90 | 2e-60 | 61/181 | [ref|YP_541631.1|](http://www.ncbi.nlm.nih.gov/entrez/query.fcgi?cmd=Retrieve&db=Protein&list_uids=91211645&dopt=GenPept&RID=GZD1BE95013&log$=protalign&blast_rank=1) prophage integrase [Escherichia coli UTI89] |
|  |  |  |  |  |  | 4e-60 | 62/177 | [ref\|YP_311283.1\|](http://www.ncbi.nlm.nih.gov/entrez/query.fcgi?cmd=Retrieve&db=Protein&list_uids=74312864&dopt=GenPept&RID=GZD1BE95013&log$=protalign&blast_rank=3) putative prophage Sf6-like integrase [Shigella sonnei Ss046] |
|  |  |  |  |  |  | 6e-60 | 60/181 | [ref\|NP_112034.1\|](http://www.ncbi.nlm.nih.gov/entrez/query.fcgi?cmd=Retrieve&db=Protein&list_uids=13559824&dopt=GenPept&RID=GZD1BE95013&log$=protalign&blast_rank=5) integrase [Enterobacteria phage HK620] |
|  |  |  |  |  |  | 6e-60 | 61/181 | [ref\|NP_958192.1\|](http://www.ncbi.nlm.nih.gov/entrez/query.fcgi?cmd=Retrieve&db=Protein&list_uids=41057294&dopt=GenPept&RID=GZD1BE95013&log$=protalign&blast_rank=6) gene 16 protein [Enterobacteria phage Sf6] |
| 18 | 1373502 1374152 | ATG TGA | 217 | - | 49.31 | 5e-69 | 61/202 | [ref|ZP_03027328.1|](http://www.ncbi.nlm.nih.gov/entrez/query.fcgi?cmd=Retrieve&db=Protein&list_uids=191165487&dopt=GenPept&RID=GZD4JM3H01R&log$=protalign&blast_rank=1) integrase [Escherichia coli B7A] |
|  |  |  |  |  |  | 9e-69 | 61/202 | [ref\|NP_958192.1\|](http://www.ncbi.nlm.nih.gov/entrez/query.fcgi?cmd=Retrieve&db=Protein&list_uids=41057294&dopt=GenPept&RID=GZD4JM3H01R&log$=protalign&blast_rank=2) gene 16 protein [Enterobacteria phage Sf6] |
|  |  |  |  |  |  | 2e-68 | 61/202 | [ref\|NP_112034.1\|](http://www.ncbi.nlm.nih.gov/entrez/query.fcgi?cmd=Retrieve&db=Protein&list_uids=13559824&dopt=GenPept&RID=GZD4JM3H01R&log$=protalign&blast_rank=3) integrase [Enterobacteria phage HK620] |
|  |  |  |  |  |  | 3e-68 | 61/202 | [gb\|ABQ88382.1\|](http://www.ncbi.nlm.nih.gov/entrez/query.fcgi?cmd=Retrieve&db=Protein&list_uids=148566107&dopt=GenPept&RID=GZD4JM3H01R&log$=protalign&blast_rank=4) integrase [Enterobacteria phage CUS-3] |
| 19 | 1334780 1335049 | ATG TGA | 90 | - | 45.56 | 3e-09 | 52/59 | [ref|NP_112036.1|](http://www.ncbi.nlm.nih.gov/entrez/query.fcgi?cmd=Retrieve&db=Protein&list_uids=13559826&dopt=GenPept&RID=GX9AFW1401R&log$=protalign&blast_rank=1) hypothetical protein HK620p03 [Enterobacteria phage HK620]  [ref|NP_958194.1|](http://www.ncbi.nlm.nih.gov/entrez/query.fcgi?cmd=Retrieve&db=Protein&list_uids=41057296&dopt=GenPept&RID=GX9AFW1401R&log$=protalign&blast_rank=1) gene 18 protein [Enterobacteria phage Sf6] |
| 20 | 1335080 1335712 | ATG TAA | 211 | - | 43.92 | 3e-122 | 100/211 | [ref|YP_215312.1|](http://www.ncbi.nlm.nih.gov/entrez/query.fcgi?cmd=Retrieve&db=Protein&list_uids=62178895&dopt=GenPept&RID=GX9K5H1K011&log$=protalign&blast_rank=1) EaC protein [Salmonella enterica subsp. enterica serovar Choleraesuis str. SC-B67] |
|  |  |  |  |  |  | 2e-109 | 89/211 | [ref\|YP_063720.1\|](http://www.ncbi.nlm.nih.gov/entrez/query.fcgi?cmd=Retrieve&db=Protein&list_uids=51236732&dopt=GenPept&RID=GX9K5H1K011&log$=protalign&blast_rank=2) EaC [Enterobacteria phage P22] |
|  |  |  |  |  |  | 1e-99 | 86/199 | [ref\|NP_720280.1\|](http://www.ncbi.nlm.nih.gov/entrez/query.fcgi?cmd=Retrieve&db=Protein&list_uids=24371540&dopt=GenPept&RID=GX9K5H1K011&log$=protalign&blast_rank=4) Eac [Enterobacteria phage ST64T] |
| 21 | 1335786 1336325 | GTG TAA | 180 | - | 48.33 | 2e-82 | 97/146 | [ref|YP_215313.1|](http://www.ncbi.nlm.nih.gov/entrez/query.fcgi?cmd=Retrieve&db=Protein&list_uids=62178896&dopt=GenPept&RID=GX9T1P8N01R&log$=protalign&blast_rank=1) Eaa protein [Salmonella enterica subsp. enterica serovar Choleraesuis str. SC-B67] |
|  |  |  |  |  |  | 1e-46 | 68/129 | [ref\|NP_059588.1\|](http://www.ncbi.nlm.nih.gov/entrez/query.fcgi?cmd=Retrieve&db=Protein&list_uids=9635497&dopt=GenPept&RID=GX9T1P8N01R&log$=protalign&blast_rank=2) EaA [Enterobacteria phage P22] |
|  |  |  |  |  |  | 6e-40 | 52/168 | [dbj\|BAA94184.1\|](http://www.ncbi.nlm.nih.gov/entrez/query.fcgi?cmd=Retrieve&db=Protein&list_uids=7649906&dopt=GenPept&RID=GX9T1P8N01R&log$=protalign&blast_rank=6) hypothetical protein [Enterobacteria phage VT2-Sakai] |
|  |  |  |  |  |  | 1e-39 | 52/168 | [ref\|NP_049538.1\|](http://www.ncbi.nlm.nih.gov/entrez/query.fcgi?cmd=Retrieve&db=Protein&list_uids=9632544&dopt=GenPept&RID=GX9T1P8N01R&log$=protalign&blast_rank=9) hypothetical protein 933Wp78 [Enterobacteria phage 933W] |
|  |  |  |  |  |  | 3e-39 | 65/120 | [ref\|NP_700404.1\|](http://www.ncbi.nlm.nih.gov/entrez/query.fcgi?cmd=Retrieve&db=Protein&list_uids=23505475&dopt=GenPept&RID=GX9T1P8N01R&log$=protalign&blast_rank=11) hypothetical protein sb31 [Salmonella phage ST64B] |
|  |  |  |  |  |  | 8e-37 | 50/190 | [ref\|NP_720282.1\|](http://www.ncbi.nlm.nih.gov/entrez/query.fcgi?cmd=Retrieve&db=Protein&list_uids=24371542&dopt=GenPept&RID=GX9T1P8N01R&log$=protalign&blast_rank=12) Eaa2 [Enterobacteria phage ST64T] |
|  |  |  |  |  |  | 2e-33 | 90/71 | [ref\|YP_224174.1\|](http://www.ncbi.nlm.nih.gov/entrez/query.fcgi?cmd=Retrieve&db=Protein&list_uids=62362249&dopt=GenPept&RID=GX9T1P8N01R&log$=protalign&blast_rank=13) gp36 [Enterobacteria phage ES18] |
| 22 | 1336325 1336903 | ATG TGA | 193 | - | 48.88 | 2e-64 | 77/163 | [ref|ZP_03217465.1|](http://www.ncbi.nlm.nih.gov/entrez/query.fcgi?cmd=Retrieve&db=Protein&list_uids=200390854&dopt=GenPept&RID=GXA5EBMB01R&log$=protalign&blast_rank=1) Eaa protein [Salmonella enterica subsp. enterica serovar Virchow str. SL491] |
|  |  |  |  |  |  | 9e-06 | 53/52 | [ref\|YP_224175.1\|](http://www.ncbi.nlm.nih.gov/entrez/query.fcgi?cmd=Retrieve&db=Protein&list_uids=62362250&dopt=GenPept&RID=GXA5EBMB01R&log$=protalign&blast_rank=6) gp37 [Enterobacteria phage ES18] |
|  |  |  |  |  |  | 0.001 | 41/53 | [ref\|NP_597893.1\|](http://www.ncbi.nlm.nih.gov/entrez/query.fcgi?cmd=Retrieve&db=Protein&list_uids=19343383&dopt=GenPept&RID=GXA5EBMB01R&log$=protalign&blast_rank=11) hypothetical protein HK022p34 [Enterobacteria phage HK022] |
|  |  |  |  |  |  | 0.002 | 45/53 | [ref\|NP_720283.1\|](http://www.ncbi.nlm.nih.gov/entrez/query.fcgi?cmd=Retrieve&db=Protein&list_uids=24371543&dopt=GenPept&RID=GXA5EBMB01R&log$=protalign&blast_rank=12) Eaa1 [Enterobacteria phage ST64T] |
|  |  |  |  |  |  | 0.049 | 35/122 | [ref\|NP_059588.1\|](http://www.ncbi.nlm.nih.gov/entrez/query.fcgi?cmd=Retrieve&db=Protein&list_uids=9635497&dopt=GenPept&RID=GXA5EBMB01R&log$=protalign&blast_rank=16) EaA [Enterobacteria phage P22] |
|  |  |  |  |  |  | 0.13 | 38/81 | [ref\|NP_112038.1\|](http://www.ncbi.nlm.nih.gov/entrez/query.fcgi?cmd=Retrieve&db=Protein&list_uids=13559828&dopt=GenPept&RID=GXA5EBMB01R&log$=protalign&blast_rank=18) hypothetical protein HK620p05 [Enterobacteria phage HK620] |
|  |  |  |  |  |  | 2.6 | 37/61 | [ref\|NP_700404.1\|](http://www.ncbi.nlm.nih.gov/entrez/query.fcgi?cmd=Retrieve&db=Protein&list_uids=23505475&dopt=GenPept&RID=GXA5EBMB01R&log$=protalign&blast_rank=35) hypothetical protein sb31 [Salmonella phage ST64B] |
| 23 | 1337110 1337922 | ATG TGA | 271 | - | 52.28 | 9e-55 | 76/148 | [ref|YP_063721.1|](http://www.ncbi.nlm.nih.gov/entrez/query.fcgi?cmd=Retrieve&db=Protein&list_uids=51236733&dopt=GenPept&RID=GXAG1X9Z01R&log$=protalign&blast_rank=5) EaD [Enterobacteria phage P22] |
|  |  |  |  |  |  | 9e-54 | 48/285 | [ref\|NP_848253.1\|](http://www.ncbi.nlm.nih.gov/entrez/query.fcgi?cmd=Retrieve&db=Protein&list_uids=30387424&dopt=GenPept&RID=GXAG1X9Z01R&log$=protalign&blast_rank=7) Ead-like protein [Enterobacteria phage epsilon15] |
|  |  |  |  |  |  | 5e-32 | 89/77 | [ref\|YP_224179.1\|](http://www.ncbi.nlm.nih.gov/entrez/query.fcgi?cmd=Retrieve&db=Protein&list_uids=62362254&dopt=GenPept&RID=GXAG1X9Z01R&log$=protalign&blast_rank=8) gp41 [Enterobacteria phage ES18] |
| 24 | 1337901 1338299 | ATG TGA | 133 | - | 49.12 | 2e-74 | 99/133 | [ref|ZP_03220544.1|](http://www.ncbi.nlm.nih.gov/entrez/query.fcgi?cmd=Retrieve&db=Protein&list_uids=204929470&dopt=GenPept&RID=GXAHV8Z801R&log$=protalign&blast_rank=1) conserved hypothetical protein [Salmonella enterica subsp. enterica serovar Javiana str. GA_MM04042433] |
| 25 | 1338678 1339154 | ATG TGA | 159 | - | 51.15 | 1e-42 | 70/136 | [ref|ZP_02682965.1|](http://www.ncbi.nlm.nih.gov/entrez/query.fcgi?cmd=Retrieve&db=Protein&list_uids=168260992&dopt=GenPept&RID=GXAKFMZE01R&log$=protalign&blast_rank=1) HNH endonuclease family protein [Salmonella enterica subsp. enterica serovar Hadar str. RI_05P066] |
|  |  |  |  |  |  | 6e-30 | 51/154 | [ref\|YP_224181.1\|](http://www.ncbi.nlm.nih.gov/entrez/query.fcgi?cmd=Retrieve&db=Protein&list_uids=62362256&dopt=GenPept&RID=GXAKFMZE01R&log$=protalign&blast_rank=4) gp43 [Enterobacteria phage ES18] |
|  |  |  |  |  |  | 4e-19 | 100/44 | [ref\|NP_720287.1\|](http://www.ncbi.nlm.nih.gov/entrez/query.fcgi?cmd=Retrieve&db=Protein&list_uids=24371547&dopt=GenPept&RID=GXAKFMZE01R&log$=protalign&blast_rank=6) Eae [Enterobacteria phage ST64T] |
|  |  |  |  |  |  | 3e-13 | 51/91 | [ref\|YP_006365.1\|](http://www.ncbi.nlm.nih.gov/entrez/query.fcgi?cmd=Retrieve&db=Protein&list_uids=46358657&dopt=GenPept&RID=GXAKFMZE01R&log$=protalign&blast_rank=9) ORF9 [Enterobacteria phage ST104] |
|  |  |  |  |  |  | 1e-09 | 37/119 | [ref\|NP_059592.1\|](http://www.ncbi.nlm.nih.gov/entrez/query.fcgi?cmd=Retrieve&db=Protein&list_uids=9635501&dopt=GenPept&RID=GXAKFMZE01R&log$=protalign&blast_rank=11) EaE [Enterobacteria phage P22] |
|  |  |  |  |  |  | 6e-07 | 31/166 | [gb\|ABQ88437.1\|](http://www.ncbi.nlm.nih.gov/entrez/query.fcgi?cmd=Retrieve&db=Protein&list_uids=148566162&dopt=GenPept&RID=GXAKFMZE01R&log$=protalign&blast_rank=16) hypothetical protein ECRS218_0057 [Enterobacteria phage CUS-3] |
|  |  |  |  |  |  | 8e-05 | 27/154 | [ref\|NP_112041.1\|](http://www.ncbi.nlm.nih.gov/entrez/query.fcgi?cmd=Retrieve&db=Protein&list_uids=13559831&dopt=GenPept&RID=GXAKFMZE01R&log$=protalign&blast_rank=21) hypothetical protein HK620p08 [Enterobacteria phage HK620] |
| 26 | 1339499 1339777 | ATG TGA | 93 | - | 32.36 | 7e-12 | 47/88 | [ref|NP_597900.1|](http://www.ncbi.nlm.nih.gov/entrez/query.fcgi?cmd=Retrieve&db=Protein&list_uids=19343390&dopt=GenPept&RID=GXAMVV0E01R&log$=protalign&blast_rank=9) putative endonuclease [Enterobacteria phage HK022] |
|  |  |  |  |  |  | 8e-12 | 47/88 | [ref\|YP_002274239.1\|](http://www.ncbi.nlm.nih.gov/entrez/query.fcgi?cmd=Retrieve&db=Protein&list_uids=209447154&dopt=GenPept&RID=GXAMVV0E01R&log$=protalign&blast_rank=10) putative HNH endonuclease [Stx2-converting phage 1717] |
| 27 | 1339954 1340244 | ATG TGA | 97 | - | 45.02 | 3e-21 | 55/97 | [ref|YP_001571676.1|](http://www.ncbi.nlm.nih.gov/entrez/query.fcgi?cmd=Retrieve&db=Protein&list_uids=161504564&dopt=GenPept&RID=GXARVDGN01R&log$=protalign&blast_rank=1) hypothetical protein SARI_02677 [Salmonella enterica subsp. arizonae serovar 62:z4,z23:--] |
|  |  |  |  |  |  | 5e-21 | 54/97 | [ref\|NP_720289.1\|](http://www.ncbi.nlm.nih.gov/entrez/query.fcgi?cmd=Retrieve&db=Protein&list_uids=24371548&dopt=GenPept&RID=GXARVDGN01R&log$=protalign&blast_rank=2) Abc2 [Enterobacteria phage ST64T] |
|  |  |  |  |  |  | 7e-21 | 54/97 | [ref\|NP_059594.1\|](http://www.ncbi.nlm.nih.gov/entrez/query.fcgi?cmd=Retrieve&db=Protein&list_uids=9635503&dopt=GenPept&RID=GXARVDGN01R&log$=protalign&blast_rank=3) Abc2 [Enterobacteria phage P22] |
|  |  |  |  |  |  | 3e-19 | 51/97 | [ref\|NP_037724.1\|](http://www.ncbi.nlm.nih.gov/entrez/query.fcgi?cmd=Retrieve&db=Protein&list_uids=9634186&dopt=GenPept&RID=GXARVDGN01R&log$=protalign&blast_rank=8) Gp39 [Enterobacteria phage HK97] |
|  |  |  |  |  |  | 2e-18 | 51/97 | [ref\|NP_037689.1\|](http://www.ncbi.nlm.nih.gov/entrez/query.fcgi?cmd=Retrieve&db=Protein&list_uids=9634147&dopt=GenPept&RID=GXARVDGN01R&log$=protalign&blast_rank=10) Abc2 protein [Enterobacteria phage HK022] |
|  |  |  |  |  |  | 4e-18 | 51/98 | [ref\|NP_112043.1\|](http://www.ncbi.nlm.nih.gov/entrez/query.fcgi?cmd=Retrieve&db=Protein&list_uids=13559833&dopt=GenPept&RID=GXARVDGN01R&log$=protalign&blast_rank=12) hypothetical protein HK620p10 [Enterobacteria phage HK620] |
|  |  |  |  |  |  | 8e-18 | 50/97 | [ref\|YP_006367.1\|](http://www.ncbi.nlm.nih.gov/entrez/query.fcgi?cmd=Retrieve&db=Protein&list_uids=46358659&dopt=GenPept&RID=GXARVDGN01R&log$=protalign&blast_rank=13) Abc2 [Enterobacteria phage ST104] |
|  |  |  |  |  |  | 3e-17 | 52/97 | [ref\|YP_224184.1\|](http://www.ncbi.nlm.nih.gov/entrez/query.fcgi?cmd=Retrieve&db=Protein&list_uids=62362259&dopt=GenPept&RID=GXARVDGN01R&log$=protalign&blast_rank=14) gp46 [Enterobacteria phage ES18] |
|  |  |  |  |  |  | 1e-16 | 51/98 | [ref\|NP_958201.1\|](http://www.ncbi.nlm.nih.gov/entrez/query.fcgi?cmd=Retrieve&db=Protein&list_uids=41057304&dopt=GenPept&RID=GXARVDGN01R&log$=protalign&blast_rank=16) gene 25 protein [Enterobacteria phage Sf6] |
| 28 | 1340265 1340810 | ATG TAA | 182 | - | 46.89 | 3e-103 | 98/182 | [ref|YP_215318.1|](http://www.ncbi.nlm.nih.gov/entrez/query.fcgi?cmd=Retrieve&db=Protein&list_uids=62178901&dopt=GenPept&RID=GXAUFP2001R&log$=protalign&blast_rank=1) endodeoxyribonuclease [Salmonella enterica subsp. enterica serovar Choleraesuis str. SC-B67] |
|  |  |  |  |  |  | 3e-54 | 58/181 | [ref\|NP_700403.1\|](http://www.ncbi.nlm.nih.gov/entrez/query.fcgi?cmd=Retrieve&db=Protein&list_uids=23505474&dopt=GenPept&RID=GXAUFP2001R&log$=protalign&blast_rank=6) Endodeoxyribonuclease [Salmonella phage ST64B] |
|  |  |  |  |  |  | 2e-50 | 55/178 | [ref\|ZP_03045860.1\|](http://www.ncbi.nlm.nih.gov/entrez/query.fcgi?cmd=Retrieve&db=Protein&list_uids=193064783&dopt=GenPept&RID=GXAUFP2001R&log$=protalign&blast_rank=9) exonuclease family protein [Escherichia coli E22] |
| 29 | 1341073 1341300 | GTG TGA | 75 | - | 50.44 | 3e-31 | 100/66 | [ref|YP_215319.1|](http://www.ncbi.nlm.nih.gov/entrez/query.fcgi?cmd=Retrieve&db=Protein&list_uids=62178902&dopt=GenPept&RID=SVWBCS2J011&log$=protalign&blast_rank=1) ssDNA-binding protein controls activity of RecBCD nuclease [Salmonella enterica subsp. enterica serovar Choleraesuis str. SC-B67] |
|  |  |  |  |  |  | 7e-26 | 78/66 | [ref\|NP_958203.1\|](http://www.ncbi.nlm.nih.gov/entrez/query.fcgi?cmd=Retrieve&db=Protein&list_uids=41057305&dopt=GenPept&RID=SVWBCS2J011&log$=protalign&blast_rank=3) gene 27 protein [Enterobacteria phage Sf6] |
|  |  |  |  |  |  | 1e-25 | 80/66 | [ref\|YP_224186.1\|](http://www.ncbi.nlm.nih.gov/entrez/query.fcgi?cmd=Retrieve&db=Protein&list_uids=62362261&dopt=GenPept&RID=SVWBCS2J011&log$=protalign&blast_rank=5) gp48 [Enterobacteria phage ES18] |
| 30 | 1341328 1342032 | ATG TAA | 235 | - | 47.94 | 1e-128 | 94/235 | [ref|NP_958204.1|](http://www.ncbi.nlm.nih.gov/entrez/query.fcgi?cmd=Retrieve&db=Protein&list_uids=41057306&dopt=GenPept&RID=GXAWPFJC01R&log$=protalign&blast_rank=3) gene 28 protein [Enterobacteria phage Sf6] |
|  |  |  |  |  |  | 1e-124 | 90/235 | [ref\|YP_224187.1\|](http://www.ncbi.nlm.nih.gov/entrez/query.fcgi?cmd=Retrieve&db=Protein&list_uids=62362262&dopt=GenPept&RID=GXAWPFJC01R&log$=protalign&blast_rank=6) gp49 [Enterobacteria phage ES18] |
|  |  |  |  |  |  | 2e-123 | 90/235 | [ref\|YP_006369.1\|](http://www.ncbi.nlm.nih.gov/entrez/query.fcgi?cmd=Retrieve&db=Protein&list_uids=46358661&dopt=GenPept&RID=GXAWPFJC01R&log$=protalign&blast_rank=8) ORF13 [Enterobacteria phage ST104] |
|  |  |  |  |  |  | 1e-122 | 88/235 | [ref\|NP_720291.1\|](http://www.ncbi.nlm.nih.gov/entrez/query.fcgi?cmd=Retrieve&db=Protein&list_uids=24371550&dopt=GenPept&RID=GXAWPFJC01R&log$=protalign&blast_rank=9) Orf-235 [Enterobacteria phage ST64T] |
|  |  |  |  |  |  | 2e-20 | 67/71 | [ref\|NP_059596.1\|](http://www.ncbi.nlm.nih.gov/entrez/query.fcgi?cmd=Retrieve&db=Protein&list_uids=9635505&dopt=GenPept&RID=GXAWPFJC01R&log$=protalign&blast_rank=20) Erf [Enterobacteria phage P22] |
| 31 | 1342162 1342383 | GTG TGA | 73 | - | 46.40 | 7e-30 | 100/62 | [ref|YP_006370.1|](http://www.ncbi.nlm.nih.gov/entrez/query.fcgi?cmd=Retrieve&db=Protein&list_uids=46358662&dopt=GenPept&RID=SVW3AX50015&log$=protalign&blast_rank=1) Kil [Enterobacteria phage ST104]  [ref|YP_215321.1|](http://www.ncbi.nlm.nih.gov/entrez/query.fcgi?cmd=Retrieve&db=Protein&list_uids=62178904&dopt=GenPept&RID=SVW3AX50015&log$=protalign&blast_rank=1) kil protein, inhibitor of host septation in Enterobacteria phage P22 [Salmonella enterica subsp. enterica serovar Choleraesuis str. SC-B67] |
|  |  |  |  |  |  | 2e-28 | 95/62 | [ref\|YP_151614.1\|](http://www.ncbi.nlm.nih.gov/entrez/query.fcgi?cmd=Retrieve&db=Protein&list_uids=56414539&dopt=GenPept&RID=SVW3AX50015&log$=protalign&blast_rank=2) hypothetical protein SPA2421 [Salmonella enterica subsp. enterica serovar Paratyphi A str. ATCC 9150] |
|  |  |  |  |  |  | 1e-19 | 70/62 | [ref\|NP_059598.1\|](http://www.ncbi.nlm.nih.gov/entrez/query.fcgi?cmd=Retrieve&db=Protein&list_uids=9635507&dopt=GenPept&RID=SVW3AX50015&log$=protalign&blast_rank=3) Kil [Enterobacteria phage P22] |
| 32 | 1342331 1342504 | ATG TAA | 57 | - | 47.70 | 2e-25 | 96/57 | [ref|YP_215322.1|](http://www.ncbi.nlm.nih.gov/entrez/query.fcgi?cmd=Retrieve&db=Protein&list_uids=62178905&dopt=GenPept&RID=SVVWBFMA015&log$=protalign&blast_rank=1) C3-like protein [Salmonella enterica subsp. enterica serovar Choleraesuis str. SC-B67] |
|  |  |  |  |  |  | 3e-22 | 96/52 | [ref\|YP_006371.1\|](http://www.ncbi.nlm.nih.gov/entrez/query.fcgi?cmd=Retrieve&db=Protein&list_uids=46358663&dopt=GenPept&RID=SVVWBFMA015&log$=protalign&blast_rank=2) C3 [Enterobacteria phage ST104] |
|  |  |  |  |  |  | 2e-19 | 94/50 | [ref\|NP_720294.1\|](http://www.ncbi.nlm.nih.gov/entrez/query.fcgi?cmd=Retrieve&db=Protein&list_uids=24371552&dopt=GenPept&RID=SVVWBFMA015&log$=protalign&blast_rank=3) C3 [Enterobacteria phage ST64T] |
|  |  |  |  |  |  | 7e-15 | 71/52 | [ref\|NP_059599.1\|](http://www.ncbi.nlm.nih.gov/entrez/query.fcgi?cmd=Retrieve&db=Protein&list_uids=9635508&dopt=GenPept&RID=SVVWBFMA015&log$=protalign&blast_rank=4) C3 [Enterobacteria phage P22] |
|  |  |  |  |  |  | 2e-08 | 65/47 | [ref\|NP_040620.1\|](http://www.ncbi.nlm.nih.gov/entrez/query.fcgi?cmd=Retrieve&db=Protein&list_uids=9626284&dopt=GenPept&RID=SVVWBFMA015&log$=protalign&blast_rank=6) antitermination protein [Enterobacteria phage lambda]  [ref\|NP_050516.1\|](http://www.ncbi.nlm.nih.gov/entrez/query.fcgi?cmd=Retrieve&db=Protein&list_uids=9633413&dopt=GenPept&RID=SVVWBFMA015&log$=protalign&blast_rank=6) cIII [Enterobacteria phage VT2-Sakai]  [ref\|NP_859191.1\|](http://www.ncbi.nlm.nih.gov/entrez/query.fcgi?cmd=Retrieve&db=Protein&list_uids=32170946&dopt=GenPept&RID=SVVWBFMA015&log$=protalign&blast_rank=6) CIII protein [Stx1 converting phage] |
|  |  |  |  |  |  | 4e-08 | 63/47 | [ref\|NP_112048.1\|](http://www.ncbi.nlm.nih.gov/entrez/query.fcgi?cmd=Retrieve&db=Protein&list_uids=13559838&dopt=GenPept&RID=SVVWBFMA015&log$=protalign&blast_rank=8) regulatory protein cIII [Enterobacteria phage HK620] |
|  |  |  |  |  |  | 9e-08 | 63/47 | [ref\|NP_049477.1\|](http://www.ncbi.nlm.nih.gov/entrez/query.fcgi?cmd=Retrieve&db=Protein&list_uids=9632483&dopt=GenPept&RID=SVVWBFMA015&log$=protalign&blast_rank=9) regulatory protein CIII [Enterobacteria phage 933W]  regulatory protein CIII [Enterobacteria phage Min27] |
|  |  |  |  |  |  | 7e-07 | 71/39 | [ref\|NP_958208.1\|](http://www.ncbi.nlm.nih.gov/entrez/query.fcgi?cmd=Retrieve&db=Protein&list_uids=41057310&dopt=GenPept&RID=SVVWBFMA015&log$=protalign&blast_rank=10) gene 32 protein [Enterobacteria phage Sf6] |
| 33 | 1342577 1342888 | ATG TGA | 104 | - | 47.44 | 4e-55 | 100/104 | [ref|NP_720295.1|](http://www.ncbi.nlm.nih.gov/entrez/query.fcgi?cmd=Retrieve&db=Protein&list_uids=24371553&dopt=GenPept&RID=GXBDW5F401R&log$=protalign&blast_rank=1) 17 [Enterobacteria phage ST64T] |
|  |  |  |  |  |  | 1e-54 | 99/104 | [ref\|YP_006372.1\|](http://www.ncbi.nlm.nih.gov/entrez/query.fcgi?cmd=Retrieve&db=Protein&list_uids=46358664&dopt=GenPept&RID=GXBDW5F401R&log$=protalign&blast_rank=2) gp17 [Enterobacteria phage ST104] |
|  |  |  |  |  |  | 3e-51 | 92/103 | [ref\|NP_059600.1\|](http://www.ncbi.nlm.nih.gov/entrez/query.fcgi?cmd=Retrieve&db=Protein&list_uids=9635509&dopt=GenPept&RID=GXBDW5F401R&log$=protalign&blast_rank=3) hypothetical protein P22gp40 [Enterobacteria phage P22] |
| 34 | 1343517 1343813 | ATG TGA | 99 | - | 47.14 | 2e-33 | 76/99 | [ref|YP_151612.1|](http://www.ncbi.nlm.nih.gov/entrez/query.fcgi?cmd=Retrieve&db=Protein&list_uids=56414537&dopt=GenPept&RID=GXBMPT8W01R&log$=protalign&blast_rank=1) hypothetical protein SPA2419 [Salmonella enterica subsp. enterica serovar Paratyphi A str. ATCC 9150] |
|  |  |  |  |  |  | 1e-21 | 58/96 | [ref\|YP_006373.1\|](http://www.ncbi.nlm.nih.gov/entrez/query.fcgi?cmd=Retrieve&db=Protein&list_uids=46358665&dopt=GenPept&RID=GXBMPT8W01R&log$=protalign&blast_rank=2) ORF18 [Enterobacteria phage ST104] |
|  |  |  |  |  |  | 1.1 | 42/54 | [ref\|NP_059602.1\|](http://www.ncbi.nlm.nih.gov/entrez/query.fcgi?cmd=Retrieve&db=Protein&list_uids=9635511&dopt=GenPept&RID=GXBMPT8W01R&log$=protalign&blast_rank=6) hypothetical protein P22gp42 [Enterobacteria phage P22] |
| 35 | 1344030 1344365 | ATG TGA | 112 | - | 48.81 | 3e-56 | 94/112 | [ref|NP_720298.1|](http://www.ncbi.nlm.nih.gov/entrez/query.fcgi?cmd=Retrieve&db=Protein&list_uids=24371556&dopt=GenPept&RID=GXBSA64901R&log$=protalign&blast_rank=1) 24 [Enterobacteria phage ST64T] |
|  |  |  |  |  |  | 3e-41 | 76/108 | [ref\|YP_224192.1\|](http://www.ncbi.nlm.nih.gov/entrez/query.fcgi?cmd=Retrieve&db=Protein&list_uids=62362267&dopt=GenPept&RID=GXBSA64901R&log$=protalign&blast_rank=4) gp54 transcription antitermination [Enterobacteria phage ES18] |
|  |  |  |  |  |  | 3e-35 | 93/79 | [ref\|NP_059605.1\|](http://www.ncbi.nlm.nih.gov/entrez/query.fcgi?cmd=Retrieve&db=Protein&list_uids=9635514&dopt=GenPept&RID=GXBSA64901R&log$=protalign&blast_rank=6) hypothetical protein P22gp46 [Enterobacteria phage P22] |
|  |  |  |  |  |  | 2e-34 | 91/79 | [ref\|YP_006377.1\|](http://www.ncbi.nlm.nih.gov/entrez/query.fcgi?cmd=Retrieve&db=Protein&list_uids=46358669&dopt=GenPept&RID=GXBSA64901R&log$=protalign&blast_rank=9) gp24 [Enterobacteria phage ST104] |
| 36 | 1344734 1345363 | ATG TAA | 210 | - | 44.6 | 3e-66 | 56/210 | [ref|ZP_03063221.1|](http://www.ncbi.nlm.nih.gov/entrez/query.fcgi?cmd=Retrieve&db=Protein&list_uids=194430884&dopt=GenPept&RID=GXBZBN3S01R&log$=protalign&blast_rank=2) repressor protein C2 [Escherichia coli B171] |
|  |  |  |  |  |  | 2e-18 | 33/217 | [gb\|ABQ88424.1\|](http://www.ncbi.nlm.nih.gov/entrez/query.fcgi?cmd=Retrieve&db=Protein&list_uids=148566149&dopt=GenPept&RID=GXBZBN3S01R&log$=protalign&blast_rank=25) prophage repressor [Enterobacteria phage CUS-3] |
|  |  |  |  |  |  | 1e-25 | 36/214 | [ref\|YP_001290702.1\|](http://www.ncbi.nlm.nih.gov/entrez/query.fcgi?cmd=Retrieve&db=Protein&list_uids=148825949&dopt=GenPept&RID=GXBZBN3S01R&log$=protalign&blast_rank=11) putative prophage repressor CI [Haemophilus influenzae PittEE] |
|  |  |  |  |  |  | 5e-18 | 32/219 | [ref\|NP_059606.1\|](http://www.ncbi.nlm.nih.gov/entrez/query.fcgi?cmd=Retrieve&db=Protein&list_uids=9635515&dopt=GenPept&RID=GXBZBN3S01R&log$=protalign&blast_rank=28) C2 [Enterobacteria phage P22] |
| 37 | 1345464 1345679 | ATG TGA | 71 | + | 45.83 | 5e-33 | 100/71 | [ref|YP_215326.1|](http://www.ncbi.nlm.nih.gov/entrez/query.fcgi?cmd=Retrieve&db=Protein&list_uids=62178909&dopt=GenPept&RID=SVUAKGJ0013&log$=protalign&blast_rank=1) regulatory protein cro (Antirepressor) [Salmonella enterica subsp. enterica serovar Choleraesuis str. SC-B67] |
|  |  |  |  |  |  | 3e-21 | 69/71 | [ref\|NP_958215.1\|](http://www.ncbi.nlm.nih.gov/entrez/query.fcgi?cmd=Retrieve&db=Protein&list_uids=41057317&dopt=GenPept&RID=SVUAKGJ0013&log$=protalign&blast_rank=2) gene 40 protein [Enterobacteria phage Sf6] |
| 38 | 1345788 1346075 | ATG TAA | 96 | + | 50.35 | 1e-08 | 39/100 | [ref|ZP_02783914.1|](http://www.ncbi.nlm.nih.gov/entrez/query.fcgi?cmd=Retrieve&db=Protein&list_uids=168758907&dopt=GenPept&RID=GXC5XV0M01R&log$=protalign&blast_rank=1) bacteriophage CII protein [Escherichia coli O157:H7 str. EC4401] |
|  |  |  |  |  |  | 2e-07 | 37/100 | [ref\|ZP_03047548.1\|](http://www.ncbi.nlm.nih.gov/entrez/query.fcgi?cmd=Retrieve&db=Protein&list_uids=193066505&dopt=GenPept&RID=GXC5XV0M01R&log$=protalign&blast_rank=3) bacteriophage CII protein [Escherichia coli E22] |
|  |  |  |  |  |  | 8e-04 | 37/88 | [gb\|ABD60131.1\|](http://www.ncbi.nlm.nih.gov/entrez/query.fcgi?cmd=Retrieve&db=Protein&list_uids=89039243&dopt=GenPept&RID=GXC5XV0M01R&log$=protalign&blast_rank=10) CII [Enterobacteria phage lambda] |
| 39 | 1346240 1346935 | ATG TGA | 232 | + | 48.71 | 2e-52 | 86/138 | [ref|YP_001648920.1|](http://www.ncbi.nlm.nih.gov/entrez/query.fcgi?cmd=Retrieve&db=Protein&list_uids=170783638&dopt=GenPept&RID=GXC7R0SS01R&log$=protalign&blast_rank=1) putative replication protein O [Enterobacteria phage Min27] |
|  |  |  |  |  |  | 1e-38 | 58/139 | [ref\|NP_599071.1\|](http://www.ncbi.nlm.nih.gov/entrez/query.fcgi?cmd=Retrieve&db=Protein&list_uids=19549026&dopt=GenPept&RID=GXC7R0SS01R&log$=protalign&blast_rank=4) replication protein [Enterobacteria phage SfV] |
|  |  |  |  |  |  | 3e-38 | 57/143 | [ref\|NP_700415.1\|](http://www.ncbi.nlm.nih.gov/entrez/query.fcgi?cmd=Retrieve&db=Protein&list_uids=23505486&dopt=GenPept&RID=GXC7R0SS01R&log$=protalign&blast_rank=5) putative replication protein [Salmonella phage ST64B] |
| 40 | 1347049 1348926 | ATG TGA | 626 | + | 51.06 | 0.0 | 96/626 | [ref|YP_224199.1|](http://www.ncbi.nlm.nih.gov/entrez/query.fcgi?cmd=Retrieve&db=Protein&list_uids=62362274&dopt=GenPept&RID=GXCAD1E901R&log$=protalign&blast_rank=2) gp61 [Enterobacteria phage ES18] |
|  |  |  |  |  |  | 0.0 | 95/371 | [ref\|YP_001648921.1\|](http://www.ncbi.nlm.nih.gov/entrez/query.fcgi?cmd=Retrieve&db=Protein&list_uids=170783639&dopt=GenPept&RID=GXCAD1E901R&log$=protalign&blast_rank=5) putative replication protein P [Enterobacteria phage Min27] |
| 41 | 1348930 1349205 | ATG TGA | 92 | + | 41.67 | 2e-39 | 84/92 | [ref|YP_224200.1|](http://www.ncbi.nlm.nih.gov/entrez/query.fcgi?cmd=Retrieve&db=Protein&list_uids=62362275&dopt=GenPept&RID=GXCWVW0Z01R&log$=protalign&blast_rank=1) gp62 [Enterobacteria phage ES18] |
| 42 | 1349282 1349866 | ATG TAA | 195 | + | 39.93 | 2e-21 | 38/160 | [ref|NP_597900.1|](http://www.ncbi.nlm.nih.gov/entrez/query.fcgi?cmd=Retrieve&db=Protein&list_uids=19343390&dopt=GenPept&RID=GXD0TWBF01R&log$=protalign&blast_rank=31) putative endonuclease [Enterobacteria phage HK022] |
|  |  |  |  |  |  | 5e-12 | 87/39 | [ref\|NP_040634.1\|](http://www.ncbi.nlm.nih.gov/entrez/query.fcgi?cmd=Retrieve&db=Protein&list_uids=9626298&dopt=GenPept&RID=GXD0TWBF01R&log$=protalign&blast_rank=69) NinB [Enterobacteria phage lambda] |
|  |  |  |  |  |  | 5e-12 | 87/39 | [ref\|YP_006386.1\|](http://www.ncbi.nlm.nih.gov/entrez/query.fcgi?cmd=Retrieve&db=Protein&list_uids=46358678&dopt=GenPept&RID=GXD0TWBF01R&log$=protalign&blast_rank=71) NinB [Enterobacteria phage ST104] |
| 43 | 1349796 1350296 | ATG TGA | 167 | + | 46.71 | 2e-60 | 79/146 | [ref|YP_002214529.1|](http://www.ncbi.nlm.nih.gov/entrez/query.fcgi?cmd=Retrieve&db=Protein&list_uids=198241951&dopt=GenPept&RID=GXD7RJJ701R&log$=protalign&blast_rank=1) putative ninB protein [Salmonella enterica subsp. enterica serovar Dublin str. CT_02021853] |
|  |  |  |  |  |  | 3e-60 | 78/146 | [ref\|NP_037743.1\|](http://www.ncbi.nlm.nih.gov/entrez/query.fcgi?cmd=Retrieve&db=Protein&list_uids=9634204&dopt=GenPept&RID=GXD7RJJ701R&log$=protalign&blast_rank=2) Gp61 [Enterobacteria phage HK97] |
|  |  |  |  |  |  | 1e-59 | 78/146 | [gb\|ABQ88417.1\|](http://www.ncbi.nlm.nih.gov/entrez/query.fcgi?cmd=Retrieve&db=Protein&list_uids=148566142&dopt=GenPept&RID=GXD7RJJ701R&log$=protalign&blast_rank=6) unknown protein [Enterobacteria phage CUS-3] |
|  |  |  |  |  |  | 1e-59 | 78/146 | [ref\|NP_040634.1\|](http://www.ncbi.nlm.nih.gov/entrez/query.fcgi?cmd=Retrieve&db=Protein&list_uids=9626298&dopt=GenPept&RID=GXD7RJJ701R&log$=protalign&blast_rank=7) NinB [Enterobacteria phage lambda] |
|  |  |  |  |  |  | 8e-59 | 77/145 | [ref\|YP_224202.1\|](http://www.ncbi.nlm.nih.gov/entrez/query.fcgi?cmd=Retrieve&db=Protein&list_uids=62362277&dopt=GenPept&RID=GXD7RJJ701R&log$=protalign&blast_rank=10) gp64 [Enterobacteria phage ES18] |
|  |  |  |  |  |  | 3e-58 | 93/115 | [ref\|NP_958225.1\|](http://www.ncbi.nlm.nih.gov/entrez/query.fcgi?cmd=Retrieve&db=Protein&list_uids=41057327&dopt=GenPept&RID=GXD7RJJ701R&log$=protalign&blast_rank=12) gene 50 protein [Enterobacteria phage Sf6] |
|  |  |  |  |  |  | 4e-58 | 77/145 | [ref\|YP_006386.1\|](http://www.ncbi.nlm.nih.gov/entrez/query.fcgi?cmd=Retrieve&db=Protein&list_uids=46358678&dopt=GenPept&RID=GXD7RJJ701R&log$=protalign&blast_rank=13) NinB [Enterobacteria phage ST104] |
|  |  |  |  |  |  | 5e-58 | 77/145 | [dbj\|BAF80769.1\|](http://www.ncbi.nlm.nih.gov/entrez/query.fcgi?cmd=Retrieve&db=Protein&list_uids=157734763&dopt=GenPept&RID=GXD7RJJ701R&log$=protalign&blast_rank=14) hypothetical protein [Enterobacteria phage P22] |
| 44 | 1350035 1350553 | GTG TGA | 175 | - | 50.87 | 3e-43 | 95/88 | [ref|YP_215330.1|](http://www.ncbi.nlm.nih.gov/entrez/query.fcgi?cmd=Retrieve&db=Protein&list_uids=62178913&dopt=GenPept&RID=GXDABY8V01R&log$=protalign&blast_rank=1) hypothetical protein SC0343 [Salmonella enterica subsp. enterica serovar Choleraesuis str. SC-B67] |
|  |  |  |  |  |  | 2e-17 | 89/48 | [ref\|NP_859380.1\|](http://www.ncbi.nlm.nih.gov/entrez/query.fcgi?cmd=Retrieve&db=Protein&list_uids=32171136&dopt=GenPept&RID=GXDABY8V01R&log$=protalign&blast_rank=2) hypothetical protein Stx2IIp133 [Stx2 converting phage II] |
| 45 | 1350614 1350907 | ATG TGA | 98 | + | 50.34 | 2e-32 | 70/96 | [ref|YP_001437710.1|](http://www.ncbi.nlm.nih.gov/entrez/query.fcgi?cmd=Retrieve&db=Protein&list_uids=156933794&dopt=GenPept&RID=GXDCDJJX01R&log$=protalign&blast_rank=1) hypothetical protein ESA_01620 [Enterobacter sakazakii ATCC BAA-894] |
| 46 | 1350907 1351299 | ATG TGA | 131 | + | 51.91 | 6e-71 | 99/131 | [ref|YP_215331.1|](http://www.ncbi.nlm.nih.gov/entrez/query.fcgi?cmd=Retrieve&db=Protein&list_uids=62178914&dopt=GenPept&RID=GXDFE7YX01R&log$=protalign&blast_rank=1) endodeoxyribonuclease RUS [Salmonella enterica subsp. enterica serovar Choleraesuis str. SC-B67] |
|  |  |  |  |  |  | 8e-69 | 95/131 | [ref\|NP_720315.1\|](http://www.ncbi.nlm.nih.gov/entrez/query.fcgi?cmd=Retrieve&db=Protein&list_uids=24371573&dopt=GenPept&RID=GXDFE7YX01R&log$=protalign&blast_rank=2) Rus [Enterobacteria phage ST64T] |
|  |  |  |  |  |  | 7e-26 | 49/116 | [ref\|NP_112065.1\|](http://www.ncbi.nlm.nih.gov/entrez/query.fcgi?cmd=Retrieve&db=Protein&list_uids=13559855&dopt=GenPept&RID=GXDFE7YX01R&log$=protalign&blast_rank=13) holiday-junction resolvase [Enterobacteria phage HK620] |
|  |  |  |  |  |  | 7e-26 | 49/116 | [ref\|NP_037749.1\|](http://www.ncbi.nlm.nih.gov/entrez/query.fcgi?cmd=Retrieve&db=Protein&list_uids=9634210&dopt=GenPept&RID=GXDFE7YX01R&log$=protalign&blast_rank=14) Gp67 [Enterobacteria phage HK97] |
|  |  |  |  |  |  | 9e-14 | 39/119 | [ref\|NP_700418.1\|](http://www.ncbi.nlm.nih.gov/entrez/query.fcgi?cmd=Retrieve&db=Protein&list_uids=23505489&dopt=GenPept&RID=GXDFE7YX01R&log$=protalign&blast_rank=61) holiday-junction resolvase [Salmonella phage ST64B] |
| 47 | 1351329 1351502 | ATG TAA | 57 | + | 48.85 | 7e-26 | 100/57 | [ref|NP_059619.1|](http://www.ncbi.nlm.nih.gov/entrez/query.fcgi?cmd=Retrieve&db=Protein&list_uids=9635528&dopt=GenPept&RID=SVT7X56Y015&log$=protalign&blast_rank=1) NinH [Enterobacteria phage P22]  [ref|NP_720316.1|](http://www.ncbi.nlm.nih.gov/entrez/query.fcgi?cmd=Retrieve&db=Protein&list_uids=24371574&dopt=GenPept&RID=SVT7X56Y015&log$=protalign&blast_rank=1) NinH [Enterobacteria phage ST64T] |
|  |  |  |  |  |  | 7e-26 | 100/57 | [ref\|YP_006393.1\|](http://www.ncbi.nlm.nih.gov/entrez/query.fcgi?cmd=Retrieve&db=Protein&list_uids=46358685&dopt=GenPept&RID=SVT7X56Y015&log$=protalign&blast_rank=2) NinH [Enterobacteria phage ST104] |
|  |  |  |  |  |  | 1e-25 | 98/57 | [ref\|YP_224209.1\|](http://www.ncbi.nlm.nih.gov/entrez/query.fcgi?cmd=Retrieve&db=Protein&list_uids=62362284&dopt=GenPept&RID=SVT7X56Y015&log$=protalign&blast_rank=3) gp71 [Enterobacteria phage ES18] |
|  |  |  |  |  |  | 3e-12 | 68/47 | [ref\|NP_040640.1\|](http://www.ncbi.nlm.nih.gov/entrez/query.fcgi?cmd=Retrieve&db=Protein&list_uids=9626304&dopt=GenPept&RID=SVT7X56Y015&log$=protalign&blast_rank=4) NinH protein [Enterobacteria phage lambda]  [ref\|NP_958231.1\|](http://www.ncbi.nlm.nih.gov/entrez/query.fcgi?cmd=Retrieve&db=Protein&list_uids=41057330&dopt=GenPept&RID=SVT7X56Y015&log$=protalign&blast_rank=4) gene 57 protein [Enterobacteria phage Sf6] |
|  |  |  |  |  |  | 1e-11 | 70/47 | [ref\|NP_049498.1\|](http://www.ncbi.nlm.nih.gov/entrez/query.fcgi?cmd=Retrieve&db=Protein&list_uids=9632504&dopt=GenPept&RID=SVT7X56Y015&log$=protalign&blast_rank=8) hypothetical protein 933Wp38 [Enterobacteria phage 933W]  [ref\|YP_794126.1\|](http://www.ncbi.nlm.nih.gov/entrez/query.fcgi?cmd=Retrieve&db=Protein&list_uids=116222071&dopt=GenPept&RID=SVT7X56Y015&log$=protalign&blast_rank=8) protein ninH [Stx2-converting phage 86]  [dbj\|BAA94138.1\|](http://www.ncbi.nlm.nih.gov/entrez/query.fcgi?cmd=Retrieve&db=Protein&list_uids=7649860&dopt=GenPept&RID=SVT7X56Y015&log$=protalign&blast_rank=8) hypothetical protein [Enterobacteria phage VT2-Sakai]  [gb\|ABY49892.1\|](http://www.ncbi.nlm.nih.gov/entrez/query.fcgi?cmd=Retrieve&db=Protein&list_uids=163955742&dopt=GenPept&RID=SVT7X56Y015&log$=protalign&blast_rank=8) hypothetical protein [Enterobacteria phage Min27] |
|  |  |  |  |  |  | 2e-09 | 62/50 | [ref\|NP_037750.1\|](http://www.ncbi.nlm.nih.gov/entrez/query.fcgi?cmd=Retrieve&db=Protein&list_uids=9634211&dopt=GenPept&RID=SVT7X56Y015&log$=protalign&blast_rank=10) Gp68 [Enterobacteria phage HK97] |
|  |  |  |  |  |  | 2e-09 | 62/50 | [ref\|NP_112066.1\|](http://www.ncbi.nlm.nih.gov/entrez/query.fcgi?cmd=Retrieve&db=Protein&list_uids=13559856&dopt=GenPept&RID=SVT7X56Y015&log$=protalign&blast_rank=12) hypothetical protein HK620p33 [Enterobacteria phage HK620] |
| 48 | 1351483 1351642 | ATG TGA | 59 | + | 53.89 | 1e-26 | 100/59 | [ref|YP_215332.1|](http://www.ncbi.nlm.nih.gov/entrez/query.fcgi?cmd=Retrieve&db=Protein&list_uids=62178915&dopt=GenPept&RID=SVT58N5S013&log$=protalign&blast_rank=1) NinZ [Salmonella enterica subsp. enterica serovar Choleraesuis str. SC-B67] |
|  |  |  |  |  |  | 1e-25 | 96/59 | [ref\|NP_720317.1\|](http://www.ncbi.nlm.nih.gov/entrez/query.fcgi?cmd=Retrieve&db=Protein&list_uids=24371575&dopt=GenPept&RID=SVT58N5S013&log$=protalign&blast_rank=2) NinZ [Enterobacteria phage ST64T] |
|  |  |  |  |  |  | 1e-24 | 94/59 | [ref\|YP_063729.1\|](http://www.ncbi.nlm.nih.gov/entrez/query.fcgi?cmd=Retrieve&db=Protein&list_uids=51236741&dopt=GenPept&RID=SVT58N5S013&log$=protalign&blast_rank=4) NinZ [Enterobacteria phage P22] |
|  |  |  |  |  |  | 2e-24 | 93/59 | [ref\|YP_224210.1\|](http://www.ncbi.nlm.nih.gov/entrez/query.fcgi?cmd=Retrieve&db=Protein&list_uids=62362285&dopt=GenPept&RID=SVT58N5S013&log$=protalign&blast_rank=5) gp72 [Enterobacteria phage ES18] |
|  |  |  |  |  |  | 4e-24 | 93/59 | [ref\|YP_006394.1\|](http://www.ncbi.nlm.nih.gov/entrez/query.fcgi?cmd=Retrieve&db=Protein&list_uids=46358686&dopt=GenPept&RID=SVT58N5S013&log$=protalign&blast_rank=6) NinZ [Enterobacteria phage ST104] |
| 49 | 1351659 1352174 | ATG TAG | 172 | + | 43.99 | 2e-97 | 99/172 | [ref|YP_215333.1|](http://www.ncbi.nlm.nih.gov/entrez/query.fcgi?cmd=Retrieve&db=Protein&list_uids=62178916&dopt=GenPept&RID=GXDJM1WG01R&log$=protalign&blast_rank=1) gp23-like protein [Salmonella enterica subsp. enterica serovar Choleraesuis str. SC-B67] |
|  |  |  |  |  |  | 1e-95 | 97/172 | [ref\|NP_720318.1\|](http://www.ncbi.nlm.nih.gov/entrez/query.fcgi?cmd=Retrieve&db=Protein&list_uids=24371576&dopt=GenPept&RID=GXDJM1WG01R&log$=protalign&blast_rank=2) gp23 [Enterobacteria phage ST64T] |
|  |  |  |  |  |  | 4e-95 | 95/172 | [ref\|YP_541660.1\|](http://www.ncbi.nlm.nih.gov/entrez/query.fcgi?cmd=Retrieve&db=Protein&list_uids=91211674&dopt=GenPept&RID=GXDJM1WG01R&log$=protalign&blast_rank=3) bacteriophage ST64T antitermination protein gp23 [Escherichia coli UTI89] |
|  |  |  |  |  |  | 9e-95 | 95/172 | [ref\|NP_958233.1\|](http://www.ncbi.nlm.nih.gov/entrez/query.fcgi?cmd=Retrieve&db=Protein&list_uids=41057332&dopt=GenPept&RID=GXDJM1WG01R&log$=protalign&blast_rank=4) gene 59 protein [Enterobacteria phage Sf6] |
|  |  |  |  |  |  | 9e-74 | 81/161 | [ref\|YP_002274250.1\|](http://www.ncbi.nlm.nih.gov/entrez/query.fcgi?cmd=Retrieve&db=Protein&list_uids=209447165&dopt=GenPept&RID=GXDJM1WG01R&log$=protalign&blast_rank=8) antiterminator Q protein [Stx2-converting phage 1717] |
| 50 | 1352638 1352958 | ATG TAA | 107 | + | 48.91 | 9e-55 | 100/107 | [ref|NP_958234.1|](http://www.ncbi.nlm.nih.gov/entrez/query.fcgi?cmd=Retrieve&db=Protein&list_uids=41057333&dopt=GenPept&RID=GXDP5FVF011&log$=protalign&blast_rank=1) gene 60 protein [Enterobacteria phage Sf6]  [gb|ABQ88408.1|](http://www.ncbi.nlm.nih.gov/entrez/query.fcgi?cmd=Retrieve&db=Protein&list_uids=148566133&dopt=GenPept&RID=GXDP5FVF011&log$=protalign&blast_rank=1) holin [Enterobacteria phage CUS-3] |
|  |  |  |  |  |  | 5e-54 | 99/107 | [ref\|NP_112068.1\|](http://www.ncbi.nlm.nih.gov/entrez/query.fcgi?cmd=Retrieve&db=Protein&list_uids=13559858&dopt=GenPept&RID=GXDP5FVF011&log$=protalign&blast_rank=2) holin [Enterobacteria phage HK620] |
|  |  |  |  |  |  | 6e-54 | 99/107 | [ref\|NP_040644.1\|](http://www.ncbi.nlm.nih.gov/entrez/query.fcgi?cmd=Retrieve&db=Protein&list_uids=9626308&dopt=GenPept&RID=GXDP5FVF011&log$=protalign&blast_rank=3) anti-holin [Enterobacteria phage lambda] |
|  |  |  |  |  |  | 4e-53 | 98/107 | [ref\|NP_037695.1\|](http://www.ncbi.nlm.nih.gov/entrez/query.fcgi?cmd=Retrieve&db=Protein&list_uids=9634153&dopt=GenPept&RID=GXDP5FVF011&log$=protalign&blast_rank=5) holin [Enterobacteria phage HK022] |
|  |  |  |  |  |  | 1e-52 | 99/105 | [ref\|YP_001551775.1\|](http://www.ncbi.nlm.nih.gov/entrez/query.fcgi?cmd=Retrieve&db=Protein&list_uids=160380505&dopt=GenPept&RID=GXDP5FVF011&log$=protalign&blast_rank=6) holin [Enterobacteria phage lambda] |
|  |  |  |  |  |  | 1e-50 | 96/107 | [ref\|NP_037752.1\|](http://www.ncbi.nlm.nih.gov/entrez/query.fcgi?cmd=Retrieve&db=Protein&list_uids=9634164&dopt=GenPept&RID=GXDP5FVF011&log$=protalign&blast_rank=7) holin [Enterobacteria phage HK97] |
|  |  |  |  |  |  | 4e-48 | 89/106 | [ref\|NP_059621.1\|](http://www.ncbi.nlm.nih.gov/entrez/query.fcgi?cmd=Retrieve&db=Protein&list_uids=9635530&dopt=GenPept&RID=GXDP5FVF011&log$=protalign&blast_rank=8) inhibitor of gene 13' protein holin [Enterobacteria phage P22]  [ref\|YP_224212.1\|](http://www.ncbi.nlm.nih.gov/entrez/query.fcgi?cmd=Retrieve&db=Protein&list_uids=62362287&dopt=GenPept&RID=GXDP5FVF011&log$=protalign&blast_rank=8) gp74 [Enterobacteria phage ES18] |
| 51 | 1352945 1353418 | ATG TGA | 157 | + | 51.05 | 5e-88 | 99/157 | [ref|YP_215336.1|](http://www.ncbi.nlm.nih.gov/entrez/query.fcgi?cmd=Retrieve&db=Protein&list_uids=62178919&dopt=GenPept&RID=GXDTS2XC013&log$=protalign&blast_rank=1) lysin [Salmonella enterica subsp. enterica serovar Choleraesuis str. SC-B67] |
|  |  |  |  |  |  | 6e-88 | 99/157 | [ref\|NP_037753.1\|](http://www.ncbi.nlm.nih.gov/entrez/query.fcgi?cmd=Retrieve&db=Protein&list_uids=9634213&dopt=GenPept&RID=GXDTS2XC013&log$=protalign&blast_rank=2) lysin [Enterobacteria phage HK97]  [ref\|NP_112069.1\|](http://www.ncbi.nlm.nih.gov/entrez/query.fcgi?cmd=Retrieve&db=Protein&list_uids=13559859&dopt=GenPept&RID=GXDTS2XC013&log$=protalign&blast_rank=2) lysozyme [Enterobacteria phage HK620] |
|  |  |  |  |  |  | 2e-87 | 98/157 | [gb\|ABQ88407.1\|](http://www.ncbi.nlm.nih.gov/entrez/query.fcgi?cmd=Retrieve&db=Protein&list_uids=148566132&dopt=GenPept&RID=GXDTS2XC013&log$=protalign&blast_rank=3) lysozyme [Enterobacteria phage CUS-3] |
|  |  |  |  |  |  | 2e-87 | 98/157 | [ref\|NP_037696.1\|](http://www.ncbi.nlm.nih.gov/entrez/query.fcgi?cmd=Retrieve&db=Protein&list_uids=9634154&dopt=GenPept&RID=GXDTS2XC013&log$=protalign&blast_rank=5) lysin [Enterobacteria phage HK022] |
|  |  |  |  |  |  | 7e-87 | 98/157 | [ref\|NP_040645.1\|](http://www.ncbi.nlm.nih.gov/entrez/query.fcgi?cmd=Retrieve&db=Protein&list_uids=9626309&dopt=GenPept&RID=GXDTS2XC013&log$=protalign&blast_rank=7) endolysin [Enterobacteria phage lambda] |
|  |  |  |  |  |  | 1e-86 | 97/157 | [ref\|NP_958236.1\|](http://www.ncbi.nlm.nih.gov/entrez/query.fcgi?cmd=Retrieve&db=Protein&list_uids=41057335&dopt=GenPept&RID=GXDTS2XC013&log$=protalign&blast_rank=8) gene 62 protein [Enterobacteria phage Sf6] |
|  |  |  |  |  |  | 3e-78 | 88/157 | [ref\|NP_599082.1\|](http://www.ncbi.nlm.nih.gov/entrez/query.fcgi?cmd=Retrieve&db=Protein&list_uids=19549037&dopt=GenPept&RID=GXDTS2XC013&log$=protalign&blast_rank=19) lysin [Enterobacteria phage SfV] |
| 52 | 1353418 1353882 | ATG TAA | 155 | + | 53.55 | 9e-73 | 87/155 | [ref|NP_112070.1|](http://www.ncbi.nlm.nih.gov/entrez/query.fcgi?cmd=Retrieve&db=Protein&list_uids=13559860&dopt=GenPept&RID=GXDWFHNV016&log$=protalign&blast_rank=5) endopeptidase [Enterobacteria phage HK620] |
|  |  |  |  |  |  | 1e-72 | 94/155 | [ref\|YP_006398.1\|](http://www.ncbi.nlm.nih.gov/entrez/query.fcgi?cmd=Retrieve&db=Protein&list_uids=46358690&dopt=GenPept&RID=GXDWFHNV016&log$=protalign&blast_rank=6) gp15 [Enterobacteria phage ST104] |
|  |  |  |  |  |  | 2e-42 | 61/154 | [ref\|NP_040646.1\|](http://www.ncbi.nlm.nih.gov/entrez/query.fcgi?cmd=Retrieve&db=Protein&list_uids=9626310&dopt=GenPept&RID=GXDWFHNV016&log$=protalign&blast_rank=41) cell lysis protein [Enterobacteria phage lambda] |
|  |  |  |  |  |  | 3e-41 | 63/149 | [ref\|YP_001700615.1\|](http://www.ncbi.nlm.nih.gov/entrez/query.fcgi?cmd=Retrieve&db=Protein&list_uids=169257238&dopt=GenPept&RID=GXDWFHNV016&log$=protalign&blast_rank=52) bacteriophage lysis protein; Rz [Phage Gifsy-1] |
| 53 | 1354098 1354781 | ATG TAA | 228 | + | 51.75 | 1e-130 | 99/228 | [ref|ZP_02682929.2|](http://www.ncbi.nlm.nih.gov/entrez/query.fcgi?cmd=Retrieve&db=Protein&list_uids=205360430&dopt=GenPept&RID=GXE0YNSC013&log$=protalign&blast_rank=1) gp79 [Salmonella enterica subsp. enterica serovar Hadar str. RI_05P066] |
|  |  |  |  |  |  | 4e-47 | 47/230 | [ref\|YP_224217.1\|](http://www.ncbi.nlm.nih.gov/entrez/query.fcgi?cmd=Retrieve&db=Protein&list_uids=62362292&dopt=GenPept&RID=GXE0YNSC013&log$=protalign&blast_rank=5) gp79 [Enterobacteria phage ES18] |
